# The 5-HT7 receptor system as a treatment target for mood and anxiety disorders: A systematic review

**DOI:** 10.1177/02698811231211228

**Published:** 2023-11-23

**Authors:** Natalie Gottlieb, Tse-Yi Li, Allan H Young, Paul RA Stokes

**Affiliations:** Department of Psychological Medicine, Institute of Psychiatry, Psychology and Neuroscience, King’s College London, London, UK

**Keywords:** 5-HT7, major depressive disorder, bipolar disorders, anxiety disorders, systematic review

## Abstract

**Background:**

Preclinical animal and preliminary human studies indicate that 5-HT7 antagonists have the potential as a new treatment approach for mood and anxiety disorders. In this systematic review, we aimed to review the relationship between the 5-HT7 receptor system and mood and anxiety disorders, and to explore the pharmacology and therapeutic potential of medications that target the 5-HT7 receptor for their treatment.

**Methods:**

Medline, Cochrane Library, EMBASE, PsycINFO databases, the National Institute of Health website Clinicaltrials.gov, controlled-trials.com, and relevant grey literature were used to search for original research articles, and reference lists of included articles were then hand searched.

**Results:**

Sixty-four studies were included in the review: 52 animal studies and 12 human studies. Studies used a variety of preclinical paradigms and questionnaires to assess change in mood, and few studies examined sleep or cognition. Forty-four out of 47 (44/47) preclinical 5-HT7 modulation studies identified potential antidepressant effects and 20/23 studies identified potential anxiolytic effects. In clinical studies, 5/7 identified potential antidepressant effects in major depressive disorder, 1/2 identified potential anxiolytic effects in generalized anxiety disorder, and 3/3 identified potential antidepressant effects in bipolar disorders.

**Conclusion:**

While there is some evidence that the 5-HT7 receptor system may be a potential target for treating mood and anxiety disorders, many agents included in the review also bind to other receptors. Further research is needed using drugs that bind specifically to 5-HT7 receptors to examine treatment proof of concept further.

## Introduction

The 5-hydroxtryptamine (5-HT or serotonin) system has been widely implicated in the pathophysiology of mood and anxiety disorders, with a main treatment focus on the serotonin transporter (SERT), 5-HT1A, 5-HT1B, and 5-HT2A receptor subtypes (Fakhoury, 2016; Thase and Denko, 2008). More recently, this has extended to the 5-HT7 receptor, which was first identified in 1993 (Bard et al., 1993; Lovenberg et al., 1993; Ruat et al., 1993). The 5-HT7 receptor is widely distributed across the brain, including the cortex, hippocampus, thalamus, and hypothalamus (Horisawa et al., 2013; Martin-Cora and Pazos, 2004; Thomas et al., 2003; To, 1995) and has been implicated in a variety of brain functions such as mood, sleep, learning and memory, stress, seizures, and circadian rhythm regulation. Moreover, the 5-HT7 receptor may have a significant role in mediating cognition, especially in people with mood disorders (Gasbarri and Pompili, 2014). Given these associations, the 5-HT7 receptor may be a promising treatment target for mood and anxiety disorders (Canese et al., 2015; Cates et al., 2013; Hedlund and Sutcliffe, 2007).

Many second-generation antipsychotic drugs currently available show high affinity to the 5-HT7 receptor and two medications with 5-HT7 antagonist properties, vortioxetine and lurasidone, have been found to enhance cognitive functioning in people with major depressive disorder (MDD) and schizophrenia (Harvey, 2015; McIntyre et al., 2013). Furthermore, preclinical animal studies have indicated the potential use of 5-HT7 antagonists for the treatment of anxiety disorders (Hedlund and Sutcliffe, 2007).

In this paper, we aimed to systematically review the relationship between 5-HT7 receptors and mood and anxiety disorders, and to further explore the pharmacology and therapeutic potential of medications that target the 5-HT7 receptor for the treatment of mood and anxiety disorders.

## Methods

The systematic review study protocol was registered on the International Prospective Register for Systematic Review (PROSPERO) database (registration number CRD42019138174). All study procedures are documented and were reported following the Preferred Reporting Items for Systematic Reviews and Meta-Analyses (PRISMA) reporting guidelines (Page et al., 2021).

### Search methods

The systematic search was conducted using Medline, Cochrane Library, EMBASE, PsycINFO, the National Institute of Health website Clinicaltrials.gov, controlled-trials.com, and relevant grey literature) from 1993 inception to March 2021. The following search string was used:

[(5-HT7 OR serotonin receptor 7 OR 5-hydroxytryptamine 7) AND (depress* OR bipolar disorder OR anxiety disorder)] OR [(5-HT7 OR serotonin receptor 7 OR 5-hydroxytryptamine 7) AND (animals OR humans OR preclinical study OR clinical trial OR experimental medicine)] OR [(5-HT7 OR serotonin receptor 7 OR 5-hydroxytryptamine 7) AND (lurasidone OR vortioxetine)] OR (5-HT7 antagonists OR 5-HT7 agonists)

Reference lists of included articles were further searched for eligible studies. If papers were not written in English, we attempted to obtain a translated version.

Systematic searches of the preselected databases were carried out independently by two researchers (TYL and NG), using the predetermined search string. Results were compiled using Rayyan QCRI software (Ouzzani et al., 2016), and titles and abstracts were independently screened by both researchers. Any studies that appeared eligible, or if there was any uncertainty about eligibility, underwent a full-text review. Final inclusion lists were compared, and any disagreements were discussed until a consensus was reached. An additional (PRS) reviewer was consulted as needed.

## Study selection

### Study and participant type

Only full-text original research articles using an appropriate control group (sham or placebo) were included. Animal studies that used a mood or anxiety disorder model and a relevant genetic or pharmacological manipulation to the 5-HT7 system were included.

For human experimental medicine studies or clinical trials, randomized control trials (RCTs) using males and females over the age of 18 fulfilling International Classification of Diseases (ICD) or Diagnostic and Statistical Manual of Mental Disorders (DSM) criteria diagnosis for an MDD, major depressive episode (MDE), bipolar affective disorder (BD) or an anxiety disorder were included. All subtypes of MDD or MDE (mild, moderate, severe, with/without psychotic features) and bipolar disorder (rapid cycling, type I, type II and other) were included. Participants who only met criteria for dysthymia or cyclothymia were excluded.

### Intervention type

Any studies that used an appropriate genetic or pharmacological manipulation to the 5-HT7 system were included in the present review. Pharmacological agents, in both preclinical and human studies, that have substantial selectivity for 5-HT7 receptors were included, such as selective agonists (AS-19, LP-44, LP-12, LP-211, E55888) (Brenchat et al., 2009; Godínez-Chaparro et al., 2011; Leopoldo et al., 2004, 2008; Sanin et al., 2004) and antagonists (SB-238719, SB-269970, SB-656104, DR-4004, DR-4446, PZ-766, JNJ-18038683, asenapine, amisulpride, imipramine, and desipramine) (Azima and Vispo, 1958; Bonaventure et al., 2012; Brodie et al., 1961; Canale et al., 2016a; Forbes et al., 2002; Frånberg et al., 2008; Hagan et al., 2000; Kikuchi et al., 1999; Puech et al., 1998; Thomas et al., 1999; Zhang et al., 2002; Zajdel et al., 2012a). Lurasidone and vortioxetine, antipsychotic and antidepressant medications, respectively, were also included due to their partial 5-HT7 receptor antagonism (Adell, 2010; Ohno et al., 1997). Studies that did not use pharmacological agents with reasonable 5-HT7 affinity were excluded.

### Outcome measures

The primary outcome measure of interest was changes in mood and anxiety behaviors after pharmacological interventions with agents that bind to, or alter, 5-HT7 receptor function. In preclinical animal studies, these behavioral changes were evaluated by use of validated functional tests that may measure depressive or anxiety symptomatology (e.g. light dark transfer test, elevated plus-maze tests, forced swim tests, and tail suspension tests). Secondary outcome measures of interests were changes in sleep or cognition measures.

For human studies, changes from baseline to endpoint in mood status, assessed by change in mood-related symptoms measured by validated rating scales such as the Hamilton Depression Rating Scale (HAM-D) or Quick Inventory of Depressive Symptomology (QIDS-CR) was the primary outcome measure (Hamilton, 1960; Rush et al., 2003). Any included data on change in sleep, cognition, or other experimental medicine markers (e.g. neuroimaging) were also collected.

## Data collection and analysis

Data extraction was conducted after individual articles were assessed against inclusion criteria and discussed further where needed. All data extraction was completed independently by two authors (NG and TYL).

### Quality measures

Study quality was measured by using the “Quality Assessment Tool for Quantitative Studies,” which was designed by Effective Public Health Practice Project (Armijo-Olivo et al., 2012; Thomas et al., 2004). Included studies were assessed in the following domains: selection bias, study design, confounders, blinding, data collection methods, withdrawal, and dropout. A global quality rating was scored independently, and any disagreements discussed by reviewers.

## Results

### Systematic search results

**Figure 1.**
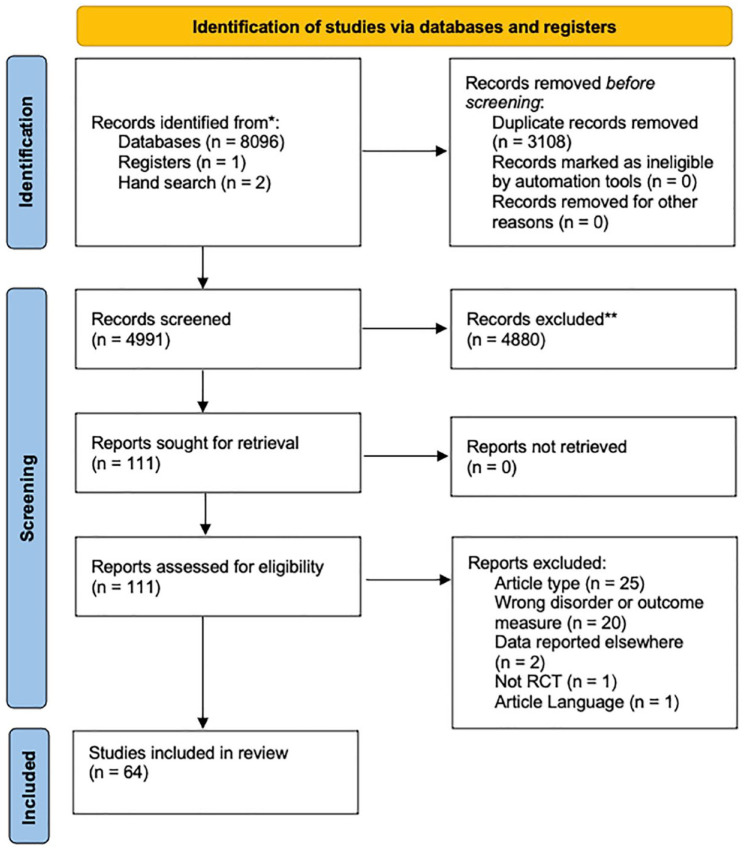
PRISMA diagram. RCT: randomized controlled trial.

The initial search identified 8097 papers. After removal of duplicates, 4989 studies underwent initial title and abstract screening leaving 109 studies for full-text review. Review of full-text articles excluded 49 articles for the following reasons: article type (7 review or meta-analysis papers and 18 conference abstracts or posters), wrong disorder or outcome measure (20), data reported elsewhere (2), not a randomized control trials study (1), and article language (1). Two further studies were added from hand searching after the original search. In total, 64 studies were included (52 animal studies and 12 human studies), and details are outlined in Figure 1.

## Preclinical study results

### Study characteristics

Fifty preclinical animal studies were included in the review. Most of the studies (48/52) examined 5-HT7 drug or knockout (KO) effects on preclinical models of depression, with a further 23 examining models of anxiety. Study details and summarized findings are listed in Table 1.

**Table 1.** Description of preclinical studies.

Author and year	Intervention	Intervention type	Evaluation	Conclusion
Abbas et al. (2009)	Amisulpride (5-HT7 KO mice)	5-HT7 receptor antagonist with multiple receptor affinity	TST/FST in mice	Decreased immobility time in WT but not KO mice, suggesting 5-HT7 receptor antagonism, and not D(2)/D(3) receptor antagonism, may underlie the antidepressant actions
Adriani et al. (2012)	LP-211, LP-378	Selective 5-HT7 receptor agonists	BWB/DL/NS in mice	Anxiolytic effects
Balcer et al. (2019)	5-HT7 WT/KO	N/A	FST/SPB/NSF/EMT in mice	Mixed results—NSF and FST showed a decrease in anxiety/depressant like responses in KO mice. EPMT did not show a difference between KO and WT. SPB (fear memory) saw more anxious behaviors in KO mice
Bonaventure et al. (2007)	SB-269970	Selective 5-HT7 receptor antagonist	TST in mice	Antidepressant effects in a variety of doses
Bonaventure et al. (2012)	JNJ-18038683	Selective 5-HT7 receptor antagonist	TST in mice; telemetry in rats	Antidepressant effects in a variety of doses, not due to overall reduction in locomotor activity
Canale et al. (2015)	Compound 32	Selective 5-HT7 receptor antagonist	FST in mice	Antidepressant effects; not due to overall reduction in locomotor activity
Canale et al. (2016a)	Compounds 17, 20, 31, 33	5-HT7 receptor antagonists; some with multiple receptor affinity	FST in mice; NOR in rats	Antidepressant effects in mice and restored drug- induced (PCP) forgetting in rats
Canale et al. (2016b)	PZ-1417, PZ-1150	Selective 5-HT7 receptor antagonists	FST/TST/FPT in mice	Antidepressant and anxiolytic effects
Canale et al. (2017)	Compounds 20, 25	Selective 5-HT7 receptor antagonist	FST/TST in mice; NOR in rats	Antidepressant effects in both FST and TST; reduced natural forgetting in rats
Cates et al. (2013)	Lurasidone	5-HT7 receptor antagonist with multiple receptor affinity	TST/FST/OSST/DL/MB in mice	Antidepressant effects (both acute and chronic models of depression) but no anxiolytic effects
Chlon-Rzepa et al. (2013)	Compounds 21, 42	5HT-7 receptor antagonist with multiple receptor affinity (5-HT1A/5-HT2A)	FST/FPT in mice	Antidepressant and anxiolytic effects in compound 21 but not 42. Both compounds showed overall reduction in overall locomotor activity
Delcourte et al. (2017)	Asenapine	5-HT7 receptor antagonist with multiple receptor affinity	FST in rats	Decreased hyperlocomotion of sleep deprived rats (antimania) but did not display antidepressant effects in depression or treatment resistant depression models
Gu et al. (2017)	Compound 8j	5-HT7 receptor antagonist with multiple receptor affinity (5-HT1A/SERT)	FST in rats	Antidepressant effects
Gu et al. (2018)	Compound 21n	5-HT7 receptor antagonist with multiple receptor affinity (5-HT1A/SERT)	FST/TST in mice	Antidepressant effects
Gu et al. (2019)	Compound 19a	5-HT7 receptor antagonist with multiple receptor affinity (5-HT1A/SERT)	FST/TST in mice	Antidepressant effects
Guilloux et al. (2013)	Vortioxetine	5-HT7 receptor antagonist with multiple receptor affinity (5-HT3/5-HT1D), 5-HT1B receptor partial agonist, 5-HT1A receptor agonist and inhibitor of SERT	OF/FST/NSF in mice	Antidepressant and anxiolytic effects following acute and repeated administration associated with increased neurogenesis
Guscott et al. (2005)	SB-258719 (5-HT7 KO mice)	Selective 5-HT7 receptor antagonist	FST in mice	Antidepressant activity of SB-258719 only showed significant decrease immobility in dark—indicating antidepressant activity may be related to circadian rhythms
Hedlund et al. (2005)	SB-269970 (5-HT7 KO mice)	Selective 5-HT7 receptor antagonist	FST/TST in mice	Untreated 5-HT7 KO and treated WT mice both showed decreased immobility—suggestive of antidepressant effects. SB-268870 had no further reduction in immobility on KO mice
Hedlund and Sutcliffe (2007)	SB-269970 (5-HT7 KO mice)	Selective 5-HT7 receptor antagonist	MB in mice	Untreated 5-HT7 KO and treated WT mice both showed decreased marble burying—suggestive of anxiolytic effects. SB-268870 had no further reduction in marble burying in KO mice
Jankowska et al. (2020)	Compound 22	5-HT7 receptor antagonist with multiple receptor affinity (5-HT1A)	FST/NOR in rats	Antidepressant effects; however also overall reduction in locomotor activity. Single administration reduced drug-induced (MK-801) memory impairment
Kim et al. (2014)	Compound 1-8	5-HT7 receptor antagonist with multiple receptor affinity	FST in mice	Antidepressant effects
Kim et al. (2016)	Compound 28	Selective 5-HT7 receptor antagonist	FST in mice	Antidepressant effects
Kołaczkowski et al. (2014)	ADN-1184	5-HT7 receptor antagonist with multiple receptor affinity		Antidepressant effects in some, but not all doses
Kucwaj-Brysz et al. (2018)	Compounds 5, 6, 7, 8	Selective 5-HT7 receptor antagonists	FST/FPT in mice	Antidepressant and anxiolytic effects across most compounds; compound 8 did not significantly increase punished crossings
Latacz et al. (2018)	MF-8	5-HT7 receptor antagonist with multiple receptor affinity	FST/FPT in mice	Antidepressant and anxiolytic effects
Lax et al. (2018)	DUQ0002I	Selective 5-HT7 receptor antagonist	EOM/DL/TST/ FST in mice	Some antidepressant (FST) and anxiolytic effects; however when injected directly to CA1 hippocampal cells anxiolytic effects did not persist
Li et al. (2013)	Vortioxetine, SB-269970, AS-19	Nonselective 5-HT7 receptor antagonist; Selective 5-HT7 antagonist; Selective 5-HT7 agonist	FST in rats	Antidepressant activity from vortioxetine but NOT SB-269970; however, hormone induced depression may not be comparable. SB-269970 added to fluoxetine showed no effect. Agonist AS-19 showed increase in immobility time, suggesting a depressive effect
Maxwell et al. (2019)	DR-4004, SB-269970	Selective 5-HT7 receptor antagonists	EMT/VCDT/TST in mice	No antidepressant effects seen in TST or anxiolytic activity in EMT and VCT
Medina et al. (2014)	Compound 6	Selective 5-HT7 receptor antagonist	FST/TST in mice	Antidepressant effects in both FST/TST
Mnie-Filali et al. (2011)	SB-269970	Selective 5-HT7 receptor antagonist	OF/FST in rats	Antidepressant activity in FST with no decrease in overall locomotor activity
Mørk et al. (2012)	Lu AA21004	5-HT7 receptor antagonist with multiple receptor affinity (5-HT3/5-HT1D), 5-HT1B receptor partial agonist, 5-HT1A receptor agonist and inhibitor of SERT	FST/CFIV in FRL and FSL rats	Antidepressant and anxiolytic effects
Partyka et al. (2017)	Compounds 16, 21	5-HT7 receptor antagonist with multiple receptor affinity (D2/5-HT1A)	FST in mice	Antidepressant effects in FST with no decrease in locomotor activity
Partyka et al. (2019)	PZ-1433, ADN-1184	Selective 5-HT7 antagonist; 5-HT7 antagonist with multiple receptor affinities (5-HT6/Monoaminergic)	FST in rats	Antidepressant activity in FST with no decrease in locomotor activity
Pytka et al. (2015)	HBK-14, HBK-15	5-HT7 receptor antagonists with multiple receptor affinities (5-HT1A/5-HT3)	FST/EMT in rats; FST/FPT in mice	Overall mixed results—antidepressant effects in both mice and rats, but only anxiolytic effects in mice. No decrease in overall locomotor function in rats or mice from either compound
Pytka et al. (2017a)	HBK-14, HBK-15	5-HT7 receptor antagonists with multiple receptor affinities (5-HT1A/5-HT3)	FST/STPA in mice	Chronic HBK-14 and (to a greater degree) HBK-15 treatment showed reduction in depressive behaviors. These changes were not explained by overall decrease in locomotor activity. HBK-15 shows potential for cognitive enhancing features
Pytka et al. (2017b)	HBK-15	5-HT7 receptor antagonist with multiple receptor affinities (5-HT1A/5-HT3)	SCT/FST/EMT in mice	HBK-15 showed prevention of anhedonic, depressive, and anxious behaviors in mice undergoing chronic mild stress (model for depression). These changes were not explained by overall decrease in locomotor activity
Pytka et al. (2018)	HBK-14, HBK-15	5-HT7 receptor antagonists with multiple receptor affinities (5-HT1A/5-HT3)	SCT/FST/EMT in mice	Both chronic and acute treatments of HB-15 showed prevention of anhedonic, depressive, and anxious behaviors in corticosterone treated mice (model for depression). These changes were not explained by overall decrease in locomotor activity
Stroth and S venningsson (2015)	SB-269970	Selective 5-HT7 receptor antagonist	FST in mice	Increased immobility in the S100B-overexpressing transgenic mice was normalized
Volk et al. (2008)	Compounds 9e’, 12d, 12e	5-HT7 receptor antagonists; some with multiple receptor affinity	VCDT in rats; DL in mice	Compound 12d may have an anxiolytic effect, with small minimal effective dosages
Volk et al. (2011)	Compounds 1a, 2a	5-HT7 receptor antagonists with multiple receptor affinity	FST/DL in mice; VCDT in rats	No antidepressant effects, Compound 2a might have an anxiolytic effect, with small minimal effective dosages
Wang et al. (2019)	Compounds 7a, 15g	5-HT8 receptor antagonists with multiple receptor affinity (5-HT1A/SERT)	FST/TST in mice	Antidepressant effect of 7a in both FST and TST, however 15g only saw antidepressant effects in FST
Waszkielewicz et al. (2015)	Compounds 2, 3, 6	5-HT7 receptor antagonists with multiple receptor affinity	TST/LA/MCT in mice	Antidepressant effects in compounds 2 and 6. Also significantly higher immobility time compared to Impipramine. No differences in locomotor activity or motor coordination
Wesolowska et al. (2006a)	SB 269970	Selective 5-HT7 receptor antagonist	VCDT/EMT in rats; FPT/FST/TST in mice	Antidepressant and anxiolytic activity, not due to change in spontaneous locomotor activity
Wesolowska et al. (2006b)	SB 269970	Selective 5-HT7 receptor antagonist	VCDT/FST in rats	Specific antidepressant and anxiolytic properties, hippocampus may be one of the neuroantaomical structures involved in these activities
Wesolowska et al. (2007)	SB 269970 + citalopram, imipramine, desipramine and moclobemide	Selective 5-HT7 receptor antagonist as an addition to other antidepressants	FST in mice	Adding 5-HT7 properties (inactive dose of SB-269970) to inactive doses of multiple types of antidepressants created a significant antidepressant-like effect. None of the medication changed spontaneous locomotor activity
Wrobel et al. (2019)	MW005, Compounds 4A, 4J	5-HT7 receptor antagonists with multiple receptor affinity	FST in mice	Only MW005 showed antidepressant effects, however this compound did not have a particularly high affinity to 5-HT7
Zagórska et al. (2015)	Compounds 8, 9	5-HT7 receptor antagonists with multiple receptor affinity (5-HT1A/D2)	FPT/FST in mice	Overall mixed results—with compound 8 showing antidepressant but not anxiolytic effects, and compound 9 showing anxiolytic but not antidepressant effects
Zagórska et al. (2016)	Compound 9	5-HT7 receptor antagonist with multiple receptor affinity (5-HT1A)	FST/FPT in mice	Compound 9 showed both anxiolytic and antidepressant properties, which did NOT stimulate spontaneous locomotor activity. Evidence for both tests showing U shaped response curves
Zajdel et al. (2011)	Compound 54	Selective 5-HT7 receptor antagonist	FST in mice	Antidepressant effects
Zajdel et al. (2012b)	Compound 36	5-HT7 receptor antagonist with multiple receptor affinity, (5-HT2A/D2)	FST in mice; EMT in rats	Antidepressant effect in mice and anxiolytic effect in rats, without decrease in overall locomotor activity
Zajdel et al. (2013)	Compounds 33, 39	5-HT7 receptor antagonists with multiple receptor affinity, (5-HT1A/5-HT2A/D2)	FST in mice	Antidepressant effects
Zajdel et al. (2015)	PZ-766, PZ-1404	Selective 5-HT7 receptor antagonists	FST/FPT in mice /NOR in rats	Both compounds displayed antidepressant effects, but only PZ-1404 displayed anxiolytic activity. Both compounds reduced drug-induced (PCP) forgetting

BWB: black and white boxes; CFIV: conditioned fear-induced vocalization; DL: dark light test; EMT: elevated maze test; EOM: elevated open maze; FPT: four plate test; FST: forced swim test; FSL: finders sensitive line rats; FRL: finders resistant line rats; KO: (gene) knockout; LA: locomotor activity; LDT: light dark transfer test; MB: marble burying; MCT: motor coordination test; N/A: not applicable; NOR: novel object recognition; NS: novelty seeking test; NSF: novelty suppressed feeding; OF: open-field test; OSST: open-space swim test; SCT: sucrose consumption test; SPB: shock probe burying; STPA: step through passive avoidance task; TST: tail suspension test; VCT: Vogel conflict test; WT: wild type.

A comprehensive listing of all relevant study results, with corresponding mood-related behavioral paradigms and mood models, comparators, individual results and *p*-values can be found in Supplemental Information—Preclinical Behavioral Results.

While all included studies used compounds or genetic manipulations that reasonably targeted 5-HT7 receptors, some compounds had higher selectivity than others.

Thirty used nonspecific 5-HT7 antagonist agents, which also have moderate to high affinity to other receptors, including other 5-HT and dopaminergic receptors, while 25 studies used compounds selective to 5-HT7 receptors.

For the purpose of this review, selective compounds are considered those which had preferential binding defined as a 5-HT7 inhibition constant (K_i_) at least fourfold less than all other receptors of interest. A complete list of compounds considered in this review, with their chemical (IUPAC) name and receptor binding profiles, can be found in Supplemental Information—Receptor Activity. Receptor bindings are described by inhibition constants (K_i_ in nanomoles) unless otherwise noted (e.g. IC50 or pK_i_ values). The Psychoactive Drug Screening Program K_i_ database was used to reference all compounds (Helmuth, 2000; Roth et al., 2000). If compounds were not listed in the database, this is noted in the Supplemental Information along with source data for receptor bindings.

### Antidepressant effects

The forced swim test (FST) and the tail suspension test (TST) are often used to evaluate antidepressant agents in preclinical rodent studies, and these tests measure immobility time as a correlate of negative mood, or hopelessness (Can et al., 2011; Yankelevitch-Yahav et al., 2015). Even though the FST and TST are not recognized as effective models of depression, they are still used for screening potential antidepressants in preclinical studies (Bourin et al., 2001; Sewell et al., 2021). Within our systematic review, 43 studies used the FST, and 15 studies used the TST.

Twenty-five studies that used compounds nonspecific to the 5-HT7 receptor identified potential antidepressant effects depression such as immobility time, in both the FST or TST (Abbas et al., 2009; Cates et al., 2013; Chlon-Rzepa et al., 2013; Gu et al., 2019, 2018, 2017; Guilloux et al., 2013; Jankowska et al., 2020; Kim et al., 2014; Kołaczkowski et al., 2014; Latacz et al., 2018; Li et al., 2013; Mørk et al., 2012; Partyka et al., 2017, 2019; Pytka et al., 2015, 2017a, 2017b, 2018; Wang et al., 2019; Waszkielewicz et al., 2015; Wróbel et al., 2019; Zagórska et al., 2015, 2016; Zajdel et al., 2013).

Furthermore, 19 studies using agents with selective 5-HT7 antagonist properties found significant improvements in potential markers of depression; for example, SB-269970 was found to decrease animals’ immobility time both in water (FST) and land (TST) (Bonaventure et al., 2012, 2007; Canale et al., 2015, 2016b, 2016a, 2017; Guscott et al., 2005; Hedlund et al., 2005; Kim et al., 2016; Kucwaj-Brysz et al., 2018; Lax et al., 2018; Medina et al., 2014; Partyka et al., 2019; Stroth and Svenningsson, 2015; Wesolowska et al., 2006a, 2006b, 2007; Zajdel et al., 2011, 2015). An additional study found decreased immobility time in 5-HT7 gene KO mice, compared to wild type (WT) mice, in the FST (Balcer et al., 2019). Lastly, one study using a highly selective 5-HT7 agonist, AS-19, reported increased immobility (i.e. more depressed-like behavior) in the FST (Li et al., 2013).

Of note, the antipsychotic medication amisulpride and antidepressants with 5-HT7 antagonist properties, such as imipramine and desipramine, were found to have potential antidepressant effects in these tests (Abbas et al., 2009; Wesolowska et al., 2007). Abbas and colleagues found that amisulpride, an antipsychotic that acts primarily as a dopaminergic receptor antagonist but also has potent 5-HT7 receptor antagonism (Abbas et al., 2009), showed potential antidepressant effects in both the TST and FST in WT mice. These effects were not seen in 5-HT7 KO mice, suggesting that activity at the 5-HT7 receptor may be specifically associated with potential antidepressant properties. Another study using citalopram, imipramine, desipramine, and moclobemide found that while low doses of these agents had no significant effect, antidepressant properties were evident once combined with SB-269970, a selective 5-HT7 receptor antagonist, suggesting that it was enhanced 5HT7 antagonism that produced potential antidepressant-like effects (Wesolowska et al., 2007).

Two studies found mixed results, each with a 5-HT7 antagonist compound showing potential antidepressant effects in the FST, but not TST (Lax et al., 2018; Wang et al., 2019). A further three studies showed no significant antidepressant effects of their tested compounds in either the TST or FST (Delcourte et al., 2017; Maxwell et al., 2019; Volk et al., 2011).

### Anxiolytic effects

In addition to modulation of depressive symptoms, there is also evidence from preclinical animal studies that 5-HT7 receptor modulation may mediate changes in measures of anxiety. A total of 23 studies measured anxiety behaviors using a variety of paradigms.

Similarly to preclinical depression studies, 11 studies found potential anxiolytic effects in animal models associated with the use of 5-HT7 antagonist agents, which also have strong to moderate affinities at other serotonergic and dopaminergic receptors (Chlon-Rzepa et al., 2013; Guilloux et al., 2013; Latacz et al., 2018; Pytka et al., 2015, 2017b, 2018; Volk et al., 2008, 2011; Zagórska et al., 2015, 2016; Zajdel et al., 2012b). Eight studies found potential anxiolytic effects when investigating selective 5-HT7 receptor antagonists (Canale et al., 2016a; Hedlund and Sutcliffe, 2007; Kucwaj-Brysz et al., 2018; Lax et al., 2018; Mnie-Filali et al., 2011; Wesolowska et al., 2006a, 2006b; Zajdel et al., 2015).

These studies used animal models of anxiety where anxiolytic effects were measured by a decrease in rodents’ motion or increase their searching behaviors in anxious environments (e.g. maintaining motionless to escape from electric shock in Four-Plate Test, increasing attempts to get food under electrical shocks in Vogel Conflict Drinking Test, or burying more marbles in their bedding in Marble Burying Test). The selective 5-HT7 antagonist, SB-269970, increased antianxiety behaviors in the Vogel Conflict Drinking Test and in the elevated plus-maze test (Wesolowska et al., 2006a). A similar finding was identified in the shock threshold test and open-field test (Wesolowska et al., 2006b). In addition, mice showed less anxious behavior in the marble burying test, which may measure obsessive-compulsive behaviors (Hedlund and Sutcliffe, 2007).

Balcer and colleagues found mixed results, with 5-HT7 gene KO mice being significantly less anxious during a novelty suppressed feeding task, but not during an elevated maze plus task (Balcer et al., 2019). Two studies found no significant anxiolytic effects associated with any compounds with 5-HT7 antagonistic properties (Cates et al., 2013; Maxwell et al., 2019). One study found that the 5-HT7 agonists, LP-211 and LP-378, improved potential anxiety-related behaviors in the light/dark test and increased exploration of black and white boxes (Adriani et al., 2012).

### Other effects

In addition to antidepressant and anxiolytic effects, 5-HT7 antagonists have been investigated in animal models of mania, sleep, and cognition. One study found that hyperlocomotion in rodents induced by sleep deprivation, a behavioral model of mania, was reduced after treatment with asenapine (Delcourte et al., 2017). Rodents treated with JNJ-18038683, a selective 5-HT7 antagonist, and 5-HT7 gene KO mice both displayed increased latency to REM sleep and decreased REM duration (Bonaventure et al., 2012; Hedlund et al., 2005). Additionally, the selective 5-HT7 antagonist SB-269970 increased REM latency, decreased REM sleep duration, and reversed increases in sleep fragmentation induced by citalopram (Bonaventure et al., 2007).

Several studies have also reported that compounds with 5-HT7 antagonist properties were associated potential pro-cognitive effects, such as reversing natural forgetting impairment in rats (Canale et al., 2017; Pytka et al., 2017a) and reversing drug-induced memory impairments (Canale et al., 2016b; Jankowska et al., 2020; Zajdel et al., 2015).

## Human study results

### Study characteristics

Twelve RCTs were included in the systematic review. Most of the studies (11) were between 6 and 8 weeks long (mean study duration 7.4 weeks); however, one study lasted for 32 weeks. Eight studies explored effects in participants with MDD, two in participants with bipolar disorder, and two in participants with generalized anxiety disorder (GAD). Only one study used a highly selective 5-HT7 antagonist (JNJ-18038683), while the others used medications that had higher affinity to other receptors, in addition to 5-HT7 receptor activity. All clinical trial details and findings are summarized in Table 2.

**Table 2.** Description of human studies.

Authorship	Study design/study length/diagnoses/drug/comparator/	Age of participants (mean ± SD)/gender (M:F or F%)/total number of participants who completed treatment	Drop-outs (including AEs and LOC, not including not randomized /not treated)*	Outcome (mood) measure/change pre versus post treatment score (mean and SD) (stat method)	Clinical outcome (CGI)/secondary outcome (sleep or cognition)
Major depressive disorder					
Bonaventure et al. (2012)	RCT/7 weeks/moderate to severe MDD/20 mg JNJ-18038683 versus 20 mg escitalopram or placebo	N/R, 218 total (71 placebo, 72 JNJ, 75 escitalopram)	N/R	MADRS/PBO **=** −13.8 JNJ **=** −15.2 ESC **=** −13.5	Neither JNJ-18038683 nor escitalopram demonstrated a significant improvement from baseline to week 7 in MADRS score. *Secondary outcome (sleep)*: JNJ-18038683 prolonged REM latency and reduced REM sleep duration in healthy controls
Alvarez et al. (2012)	RCT/6 weeks/MDD/5 or 10 mg Lu AA21004 versus 225 mg venlafaxine XR or placebo	43.3 ± 11.5, 62.70%, 360 (87 placebo, 93 venlafaxine XR, 98 LU AA21004 5 mg, 82 LU AA21004 10 mg)	PBO = 18—4 AEs, 6 LoE, 8 other VEN = 20—16 AEs, 2 LoE, 2 other, LUA5 = 10—3 AEs, 6 LoE, 1 other, LUA10 = 18—7AEs, 3 LoE, 8 other	MADRS/PBO = −16.6 ± 1.0, VEN = −24.2 ± 0.9, LUA5 = −22.3 ± 0.9, LUA10 = −23.4 ± 1.0 (OC; ANCOVA)	Lu AA21004 was statistically significantly superior to PBO in mean change from baseline in MADRS total score at week 6 (*p* < 0.0001), corresponding to a standardized effect size of 0.56 (5 mg) and 0.54 (10 mg)
Henigsberg et al. (2012)	RCT/8 weeks/MDD/1, 5, or 10 mg Lu AA21004 versus placebo	46.4 ± 12.1, 62.60%, 505 total (127 placebo, 127 LUA 1 mg, 129 LUA 5 mg, 122 LUA 10 mg)	PBO = 13—2 AES, 8 LoE, 3 other, LUA1 = 13—3 AEs, 4 LoE, 6 other, LUA5 =v11—1 AE, 2 LoE, 8 other, LUA10 = 18—5 AEs, 3 LoE, 10 other	HAM-D24/PBO = −10.1 ± 0.7, LUA1 = −14.82 ± 0.7. LUA5 = −15.42 ± 0.7, LUA10 = −16.23 ± 0.8 (MMRM)	Lu AA21004 10 mg there was a significant reduction in HAM-D24 total score compared with placebo in all doses of LuAA21004 (all *p* < 0.001), corresponding to a standardized effect size of 0.37 (1 mg), 0.41 (5 mg), and 0.54 (10 mg)
Jacobsen et al. (2015)	RCT/8 weeks/MDD/10 or 20 mg Vortioxetine versus placebo	42.8 ± 12.2, 72.51%, 457 (155 placebo, 154 Vortioxetine 10 mg, 148 Vortioxetine 20 mg)	PBO = 18 -2 AE, 1 LoE, 15 other, VOR10 = 31—9 AE, 3 LoE, 19 other, VOR20 = 28—7 AE, 1 LoE, 20 other	MADRS/PBO = −10.8 ± 0.81, VOR10 = −13.0 ± 0.83, VOR20 = −14.4 ± 0.85	Vortioxetine at 20 mg showed significant improvement in MADRS score at 8 weeks compared to placebo (*p* = 0.002). The difference between placebo and 10 mg Vortioxetine did not reach significance (*p* = 0.058)
Mahableshwarkar et al. (2013)	RCT/8 weeks/MDD/2.5 or 5 mg Vortioxetine versus 60 mg duloxetine or placebo	N/R, N/R, 611 randomized	N/R	HAM-D24/*(mean and SE)*, PBO = −10.5 ± 0.76, VOR2.5 = −12.04 ± 0.74, VOR5 = −11.08 ± 0.74, DUL = −13.47 ± 0.75	Vortioxetine were associated with declines in HAM-D24 total scores compared to placebo but were not statistically significant
Mahableshwarkar et al. (2015)	RCT/8 weeks/MDD/10 or 15 mg Vortioxetine versus placebo	45.1 ± 12.4/70.15%/434 (149 placebo, 143 VOR 10 mg, 142 VOR 15 mg)	PBO = 27—6 AE, 4 LoE, 17 other, VOR10 = 26—8 AE, 2 LoE, 16 other VOR15 = 31—12 AE, 0 LoE, 19 other	MADRS/PBO = −12.87, VOR10 = −13.66, VOR15 = −13.36	Differences from placebo in primary endpoint (change in MADRS score) were not statistically significant for either 10 or 15 mg Vortioxetine. *Secondary Outcome (cognition)*: Both vortioxetine doses showed numerically greater improvement in CPFQ (cognitive and physical functioning questionnaire) scores compared to placebo, but neither were statistically significant
McIntyre et al. (2014)	RCT/8 weeks/MDD/10 or 20 mg Vortioxetine versus placebo	45.7 ± 12.0/66.22%/598 (196 placebo, 195 VOR 10 mg, 207 VOR 20 mg)	N/R	MADRS/PBO = −10.9 ± 0.6, VOR10 = −15.6 ± 0.6, VOR20 = −17.6 ± 0.6 (MMRM)	Patients on vortioxetine separated from placebo in depressive symptomology on MADRS at both 10 mg (*p* < 0.001) and 20 mg (*p* < 0.001). *Secondary Outcome (cognition)*: Patients also reported objective and subjective measures of cognitive function independent of its effect on improving depression symptoms. Both doses showed improved composite cognition scores compared to placebo (*p* < 0.001)
Generalized anxiety disorder					
Baldwin et al. (2012)	Open label + double-blinded period (20 weeks plus 24-56 weeks), GAD (DSM-IV-TR), 5 or 10 mg LU AA21004 (as decided in open-label period) versus placebo	43.3 ± 13.3, 63.20%, 278 (121 placebo, 157 LuAA21004)	N/R	HAM-A//time to relapse	Result of the primary efficacy analysis (Cox model, *p* < 0.0001) showed a statistically significant effect on the time to relapse in the double-blind period when comparing LuAA21004 to placebo. Cox proportional hazard model yielded a hazard ratio (HR) of 2.71 (*p* < 0.0001) meaning the risk of relapse for the patients in the placebo group was almost three times that for patients in the Lu AA21004 group. Lastly, proportion of patients who relapsed was significantly lower in the Lu AA21004 group than that in the placebo group (*p* < 0.0001)
Rothschild 2012	RCT/8 weeks/GAD (DSM-IV-TR), 5 mg Vortioxetine	41.2 ± 13.4, 65.80%, 239 total (125 Vortioxetine, 114 placebo)	PBO = 37—4 AEs, 3 LoE, 30 other, VOR5 = 27—3 AEs, 1 LoE, 23 other	HAM-A/PBO = −13.16 ± 0.655, VOR5 = −12.57 ± 0.646	There was no significant difference between change in HAM-A scores between Vortioxetine and placebo
Bipolar disorder					
El-Mallakh et al. (2020)	RCT (subset of larger study), 8 weeks, bipolar depression, 5–20mg Asenapine versus placebo	(18–55), N/R, 9 (4 asenapine, 5 placebo)	N/R	MADRS/PBO = −3.80 ± 9.01, ASE = −19.80 ± 8.59, HAM-A/PBO = −2.80 ± 7.95, ASE = −15.40 ± .15	Improvement in MADRS (*p* = 0.021) and HAM-A (*p* = 0.023) scores were statistically significantly greater in the Asenapine group than the placebo group measured as changes from baseline to week 8.
Loebel et al. (2014)	RCT/6 weeks/20–60 mg or 80–120 mg Lurasidone daily versus placebo in BD	N/R, 56.90%, 374 (127 placebo, 123 Lur20–60, 124 Lur80–120)	PBO = 43—11 AEs, 13 LoE, 19 other, Lur20–60 = 43—11 AEs, 12 LoE, 20 other Lur80–120 = 45—10 AEs, 5 LoE, 30 other	MADRS/PBO = −10.7 Lur20–60 = −15.4 Lur80–120 = −15.4	Lurasidone treatment significantly reduced mean MADRS total scores at week 6 for both the 20–60 mg/day group (*p* < 0.001; effect size = 0.51) and the 80–120 mg/day group (*p* < 0.001; effect size = 0.51)
Suppes et al. (2016)	RCT/6 weeks/MDD with 2–3 manic/hypomanic symptoms, 20–60 g Lurasidone versus placebo	44.9 ± 12.1, 69.38%, 189 (102 Lurasidone, 87 placebo)	PBO = 15—5 AE, 4 LoE, 6 other, LUR = 7—3 AE, 2 LoE, 2 other	MADRS/PBO = −13.0 ± 1.0, LUR = −20.5 ± 1.0	Lurasidone significantly improved depressive symptoms and overall illness severity assessed by least squares mean change at week 6 in MADRS (*p* < 0.001; effect size = 0.80) and CGI-S (*p* < 0.001; effect size = 0.60). Significant improvement in manic symptoms assess by YMRS was also observed (*p* < 0.001)

AE: adverse event; ASE: asenapine; ANCOVA: analysis of covariance; CGI(-S): clinical global impression (severity scale); DUL: duloxetine; ESC: escitalopram; GAD: generalized anxiety disorder; HAM-A: Hamilton anxiety rating scale; HAM-D24: Hamilton depression rating scale, 24 Item; JNJ: JNJ-18038683; LoC: lack of completion; LoE: lack of efficacy; LUA: LuAA21004; LUR: lurasidone; MADRS: montgomery–Åsberg depression rating scale; MMRM: mixed model for repeated measures; MDD: major depressive disorder; N/R: not reported; OC: observed cases; PBO: placebo; RCT: randomized controlled trial; SD: standard deviation; SE: standard error; VEN: venlafaxine; VOR: vortioxetine; YMRS: young mania rating scale.

### Major depressive disorder

Six studies investigated the effect of vortioxetine (LU AA21004), a medication with 5-HT1A, 5-HT1B, 5-HT3, and 5-HT7 receptors and 5-HT transporter affinities, on MDD symptoms. All studies administering vortioxetine at 20 mg found significant improvements in mood as measured by Montgomery–Åsberg Depression Rating Scale (MADRS) (Montgomery and Asberg, 1979) between baseline and week 8 (Jacobsen et al., 2015; McIntyre et al., 2014). Three studies found significant differences in mood at a 10-mg dose (Alvarez et al., 2012; Henigsberg et al., 2012; McIntyre et al., 2014); however, two only found a trend toward significance at this dosage (Jacobsen et al., 2015; Mahableshwarkar et al., 2015). Lower doses of vortioxetine (5 mg or less) found no significant changes associated with treatment (Alvarez et al., 2012; Henigsberg et al., 2012; Mahableshwarkar et al., 2013). It is important to emphasize that vortioxetine is not a 5-HT7-specific agent, and vortioxetine also blocks 5-HT1D and 5HT-3 receptors and stimulates 5-HT1A and 5HT1B receptors.

Only one study examined change in depressive symptoms after 7 weeks of 20-mg JNJ-18038683, a highly selective 5-HT7 receptor antagonist, compared to placebo and escitalopram 20 mg (Bonaventure et al., 2012). Although JNJ-18038683 did not significantly decrease depression symptoms compared to placebo, escitalopram (20 mg) also did not significantly change mood scores in this study.

### Anxiety disorders

Two studies examined the effects of medications with 5-HT7 antagonistic properties in participants with GAD. Rothschild and colleagues measured anxiety symptoms using the Hamilton Anxiety Scale (HAM-A) (Hamilton, 1959) before and after 8 weeks of treatment with 5 mg vortioxetine, but found no differences compared to placebo (Rothschild et al., 2012). Baldwin and colleagues investigated the effect of either 5 or 10 mg of vortioxetine on time to relapse in participants with GAD. At the end of a 20-week open-label treatment period, participants who responded were then randomized to 24–56 weeks of a double-blind treatment of vortioxetine (*n* = 229) or placebo (*n* = 230). The study found a statistically significant effect of vortioxetine relative to the placebo in time to relapse (Baldwin et al., 2012).

### Bipolar disorders

Lurasidone and asenapine are both atypical antipsychotics often used to treat schizophrenia and bipolar disorders, and they both have moderate affinity to 5-HT7 receptors. Lurasidone is a dopaminergic D2 and D3 receptor, 5-HT2A, 5-HT7, and α2C-adrenergic receptor antagonist, and a partial 5-HT1A agonist (Bawa and Scarff, 2015). In one study investigating bipolar depression, participants treated with lurasidone (at both 20–60 mg and 80–120 mg doses) experienced a significant improvement in MADRS scores after 6 weeks compared to placebo (Loebel et al., 2014). One study, with a sample size of nine participants, found significant improvements in both depressive and anxiety symptoms in those with bipolar depression after 8-week treatment with asenapine (El-Mallakh et al., 2020). In participants with MDD with mixed features, who experienced least 2–3 manic or hypomanic episodes, depressive symptoms measured by MADRS and manic symptoms measured by the Young Mania Rating Scale (YMRS) improved after 6 weeks of treatment of 20–60 mg lurasidone (Suppes et al., 2016; Young et al., 1978).

### Other effects

As with rodent studies, human clinical trials have observed effects of 5-HT7 receptor modulation outside of mood symptoms. McIntyre and colleagues found that both 10- and 20-mg vortioxetine significantly improved participants’ composite cognition scores compared to placebo using a predefined efficacy analysis. Additionally, they also found significant improvements in most secondary objectives and subjective patient-reported cognitive measures (McIntyre et al., 2014). Another study measuring cognitive impairment using the Cognitive and Physical Functioning Questionnaire (CPFQ) found while both 10- and 15-mg vortioxetine numerically improved scores, these improvements were not statistically significant versus placebo (Fava et al., 2009; Mahableshwarkar et al., 2015).

Only one study in the systematic review examined changes in sleep in human participants. JNJ-18038683, a specific 5HT-7 antagonist, was found to prolong REM latency and reduced REM sleep duration in healthy participants, and enhanced REM sleep suppression induced by citalopram (Bonaventure et al., 2012).

## Quality assessment

Overall, the clinical trials included in this systematic review were assessed to be between strong and moderate quality using the Quality Assessment Tool for Quantitative Studies, and the scores are listed in Table 3. All studies were considered to have a strong study design and strong blinding strategies. Some studies did not describe participant withdrawals and drop out, which is vital to a well described trial. Full quality assessment results for each study can be found in Table 3.

**Table 3.** Results of quality assessment.

Authorship	Selection bias	Study design	Confounders	Blinding	Data collection method	Withdrawals	Global rating
Alvarez et al. (2012)	1	1	1	1	1	1	Strong
Baldwin et al. (2012)	1	1	1	2	1	2	Strong
Bonaventure et al. (2012)	1	1	1	2	1	1	Strong
El-Mallakh et al. (2020)	2	1	1	2	1	1	Strong
Henigsberg et al. (2012)	2	1	1	1	1	1	Strong
Jacobsen et al. (2015)	1	1	1	1	1	1	Strong
Loebel et al. (2014)	1	1	1	2	1	1	Strong
Mahableshwarkar et al. (2013)	1	1	1	1	1	1	Strong
Mahableshwarkar et al. (2015)	1	1	1	2	1	1	Strong
McIntyre et al. (2014)	1	1	1	1	1	1	Strong
Rothschild et al. (2012)	1	1	1	2	1	1	Strong
Suppes et al. (2016)	1	1	1	1	1	1	Strong

1 = strong; 2 = moderate; 3 = weak.

## Discussion

Overall, the animal studies and human clinical trials included in this review provide preliminary evidence that 5-HT7 antagonists may potentially be useful for the treatment of mood and anxiety disorders, with 49 animal studies and 11 human studies reporting statistically significant changes in mood or behavior using 5-HT7-related pharmacological interventions. However, this review highlights that the evidence base is constrained by a lack of studies using specific 5-HT7 receptor agents, which do not influence dopaminergic or other 5-HT receptor subtypes. While many pharmacological interventions with activity at the 5-HT7 receptor have been found to induce significant changes in mood symptoms in clinical trials, these results are difficult to interpret due to the lack of 5-HT7 receptor specificity.

Preclinical studies report an improvement in depression-related (43 studies) and anxiety-related (21 studies) behaviors, further supporting that the notion that 5-HT7 receptor modulation may impact mood- and anxiety-related symptoms. For example, the selective 5-HT7 antagonist SB-269970 was associated with specific anxiolytic effects in the Vogel conflict drinking, elevated plus-maze, shock threshold, and open-field tests (Wesolowska et al., 2006b), without influencing gross locomotion. Selective 5-HT7 antagonists have also been shown to induce possible antidepressant-like behaviors in preclinical animal models (Bonaventure et al., 2007, 2012; Canale et al., 2015, 2016a, 2016b, 2017; Guscott et al., 2005; Hedlund et al., 2005; Kim et al., 2016; Kucwaj-Brysz et al., 2018; Lax et al., 2018; Medina et al., 2014; Partyka et al., 2019; Stroth and Svenningsson, 2015; Wesolowska et al., 2006a, 2006b, 2007; Zajdel et al., 2011, 2015) although the interpretation of these results is complicated by concerns over the validity of these tests measuring depressive symptoms and lack of validation of receptor binding profiles for many of the compounds used. The use of compounds with multiple receptor subtype affinity (such as MF-8, HBK-14, and HBK-15) was also associated with potential antidepressant-like and anxiolytic-like effects, although the lack of specificity of these compounds for the 5-HT7 receptor makes interpretation of these results difficult. For example, Wrobel and colleagues only found that MW005 may have antidepressant-related effects in animal models. However, this compound had lower 5-HT7 receptor selectivity compared to the newer compounds investigated in this study, which were not associated with antidepressant-related effects (Wróbel et al., 2019).

In addition to 5-HT7 antagonism, 5-HT7 agonists were found to impact emotion in preclinical animal models. One study found that 5-HT7 agonist AS-19 exacerbated depressive symptoms in rodent models of progesterone withdrawal, which are believed to model hormonally induced mood disorders in women (Li et al., 2013). Conversely, Adriani and colleagues found that LP-211 and LP-378, two 5-HT7 agonists, were associated with increased disinhibition across a variety of tasks, including B/W boxes, dark/light, and novelty seeking tasks. The researchers observed pro-locomotor and pro-exploratory behaviors, with mice spending more time in the aversive light side and white boxes (Adriani et al., 2012). Further studies using 5-HT7 agonists are needed to clarify the mechanisms underlying these observations.

In human clinical trials, several studies found an improvement of symptoms in people with MDD after treatment with vortioxetine. All studies administering vortioxetine at 20 mg found significant improvements in mood (Jacobsen et al., 2015; McIntyre et al., 2014). Those using lower dosages had more mixed results, suggesting it may be only doses of vortioxetine greater than 10 mg are effective in symptom improvement. Lastly, two studies also found improvements in cognition with use of at least 10-mg vortioxetine in these participants with MDD (Mahableshwarkar et al., 2015; McIntyre et al., 2014). As only these two studies measured changes in cognition, further research in needed.

In people with GAD, 5-mg vortioxetine was associated with no differences in anxiety symptoms (Rothschild et al., 2012); however, vortioxetine at 5 and 10 mg did show a statistically significant effect of vortioxetine relative to the placebo in time to relapse in a longer 56-week study (Baldwin et al., 2012). Taken together, these studies highlight the importance of dosage and treatment duration when considering efficacy in the treatment of affective symptoms.

In the only human trial using a selective 5-HT7 antagonist, Bonaventure and colleagues found that while there was no significant decrease in depressive symptoms compared to placebo, JNJ-18038683 prolonged REM latency and reduced REM sleep duration in healthy controls. It is important to note that in this study, neither escitalopram (20 mg), a commonly used antidepressant, nor JNJ-18038683 significantly decreased depressive symptomology as measured by the MADRS compared to placebo (Bonaventure et al., 2012).

The quality of the clinical trials included in this review was considered to be strong or moderate overall. However, one study had small sample size (*n* = 9) due to it being a subset of a larger study that was terminated early by the sponsor for non-safety-related issues (El-Mallakh et al., 2020). Quality assessments were not carried out on preclinical rodent studies as the availability of standardized quality assessment for use in animal studies is lacking (Zeng et al., 2015).

## Limitations and future directions

Despite the evidence for the potential role of the 5-HT7 receptor subtype in mood and anxiety disorders, this systematic review has several limitations.

While all the studies included in this review used pharmacological treatments with moderate to strong affinity to 5-HT7 receptor subtypes, many also have affinity to other receptor types including the SERT and 5-HT1A receptors, which are often targets for antidepressants and anxiolytics (Artigas, 2013; Nautiyal and Hen, 2017). Only 21 of the original 52 preclinical studies measuring depressive symptomology used selective 5-HT7 pharmacological interventions. While the compounds described in this systematic review were defined as selective based on a preferential binding to 5-HT7 receptors compared to others, some of these compounds did have moderate affinities to other receptors with inhibition constants less than 100 nm. For example, while PZ-766 is a potent 5-HT7 antagonist, with a K_i_ value of 0.3 nm, there is also indication of activity at the D2 receptor (K_i_ = 52 nm) (Zajdel et al., 2015). This may imply that changes in mood symptoms result in part from the modulation of SERT, 5-HT1A, or 5-HT2 receptor function. JNJ-18038683 was the only highly selective 5-HT7 antagonist used in human trials, highlighting that further research is required using more selective 5-HT7 compounds to better characterize the underlying pharmacology of the observed changes in mood and anxiety disorders.

Although antidepressant- and anxiolytic-related effects were widely examined, only one preclinical study and one clinical trial explored effects on mania-related measures using agents with wider therapeutic targets (Delcourte et al., 2017; Suppes et al., 2016). Sleep and cognition were examined as secondary outcome measures in nine animal studies and three human studies (Adriani et al., 2012; Bonaventure et al., 2012, 2007; Canale et al., 2017, 2016b; Jankowska et al., 2020; Pytka et al., 2017a; Zajdel et al., 2015). We would suggest that further research using more selective 5-HT7 compounds is necessary to fully understand the effects of 5-HT7 receptor modulation on mania, sleep, and cognition function (Gasbarri and Pompili, 2014).

Finally, it is important to note that no unpublished data were identified to be included in this systematic review, which may indicate that negative findings could have been underreported.

## Conclusions

This systematic review examined evidence for the 5-HT7 receptor as a therapeutic target for mood and anxiety disorders with 49 preclinical and 11 human studies demonstrating antidepressant- or anxiolytic-related effects using compounds that had at least moderate 5-HT7 receptor affinity. The 5-HT7 receptor may also be a potential target for the treatment of sleep disturbances or cognitive impairment associated with mood disorders, but further research is warranted. We would suggest that further studies investigate pharmacological agents with more distinct selectivity to the 5-HT7 receptor subtype, to ensure that effects are not related to their affinity to other receptors (such as 5-HT1A, D2, or SERT). Additionally, more research is needed to observe changes in cognition and sleep.

## Supplemental Material

sj-docx-1-jop-10.1177_02698811231211228 – Supplemental material for The 5-HT7 receptor system as a treatment target for mood and anxiety disorders: A systematic review Click here for additional data file. Supplemental material, sj-docx-1-jop-10.1177_02698811231211228 for The 5-HT7 receptor system as a treatment target for mood and anxiety disorders: A systematic review by Natalie Gottlieb, Tse-Yi Li, Allan H Young and Paul RA Stokes in Journal of Psychopharmacology

sj-docx-2-jop-10.1177_02698811231211228 – Supplemental material for The 5-HT7 receptor system as a treatment target for mood and anxiety disorders: A systematic review Click here for additional data file. Supplemental material, sj-docx-2-jop-10.1177_02698811231211228 for The 5-HT7 receptor system as a treatment target for mood and anxiety disorders: A systematic review by Natalie Gottlieb, Tse-Yi Li, Allan H Young and Paul RA Stokes in Journal of Psychopharmacology

sj-xlsx-3-jop-10.1177_02698811231211228 – Supplemental material for The 5-HT7 receptor system as a treatment target for mood and anxiety disorders: A systematic review Click here for additional data file. Supplemental material, sj-xlsx-3-jop-10.1177_02698811231211228 for The 5-HT7 receptor system as a treatment target for mood and anxiety disorders: A systematic review by Natalie Gottlieb, Tse-Yi Li, Allan H Young and Paul RA Stokes in Journal of Psychopharmacology
