# Supplementary material for: The 5-HT7 receptor system as a treatment target for mood and anxiety disorders: A systematic review
Source: J Psychopharmacol. 2023 Nov 23;37(12):1167–81. doi: 10.1177/02698811231211228 (PMC10714716; doi:10.1177/02698811231211228)
Supplement: sj-docx-1-jop-10.1177_02698811231211228 – Supplemental material for The 5-HT7 receptor system as a treatment target for mood and anxiety disorders: A systematic review [file sj-docx-1-jop-10.1177_02698811231211228.docx]

| **No.** | **Author / Year** | **Compound Name** | **Chemical Name** | **Groups and/or Comparator** | **Tests** | **Model** |
| --- | --- | --- | --- | --- | --- | --- |
| 1 | Abbas 2009 | Amisulpride | 4-amino-N-[(1-ethylpyrrolidin-2-yl)methyl]-5-ethylsulfonyl-2-methoxybenzamide | 5-HT7+/+ (WT) mice vs 5-HT7-/- (KO) mice; vehicle | TST | depression |
| 1 | Abbas 2009 | Amisulpride | 4-amino-N-[(1-ethylpyrrolidin-2-yl)methyl]-5-ethylsulfonyl-2-methoxybenzamide | 5-HT7+/+ (WT) mice vs 5-HT7-/- (KO) mice; vehicle | FST | depression |
| 2 | Adriani 2012 | LP-211 | N-(4-cyanophenylmethyl)-4-(2-diphenyl)-1-piperazinehexanamide | saline vehicle comparator | BWB | locomotion / exlporation |
| 2 | Adriani 2012 | LP-211 | N-(4-cyanophenylmethyl)-4-(2-diphenyl)-1-piperazinehexanamide | saline vehicle comparator | D/L | anxiety |
| 2 | Adriani 2012 | LP-211 | N-(4-cyanophenylmethyl)-4-(2-diphenyl)-1-piperazinehexanamide | saline vehicle comparator | NS | curiosity |
| 2 | Adriani 2012 | LP-378 | N-(4-Trifluorophenylmethyl)-4-(2-diphenyl)-1-piperazinehexanamide | saline vehicle comparator | BWB | locomotion / exlporation |
| 3 | Balcer 2019 | N/A | N/A | 5-HT7+/+ (WT) mice vs 5-HT7-/- (KO) mice | SPB | anxiety + cognition |
| 3 | Balcer 2019 | N/A | N/A | 5-HT7+/+ (WT) mice vs 5-HT7-/- (KO) mice | NSF | anxiety |
| 3 | Balcer 2019 | N/A | N/A | 5-HT7+/+ (WT) mice vs 5-HT7-/- (KO) mice | EMT | anxiety |
| 3 | Balcer 2019 | N/A | N/A | 5-HT7+/+ (WT) mice vs 5-HT7-/- (KO) mice | FST | depression |
| 4 | Bonaventure 2007 | SB-269970 | (2R)-1-[(3-hydroxyphenyl)sulfonyl]-2-[2-(4-methyl-1-piperidinyl)ethyl]-pyrrolidine | vehicle | TST | depression |
| 4 | Bonaventure 2007 | SB-269970 | (2R)-1-[(3-hydroxyphenyl)sulfonyl]-2-[2-(4-methyl-1-piperidinyl)ethyl]-pyrrolidine | vehicle | TST | depression |
| 4 | Bonaventure 2007 | SB-269970 | (2R)-1-[(3-hydroxyphenyl)sulfonyl]-2-[2-(4-methyl-1-piperidinyl)ethyl]-pyrrolidine | vehicle | TST | depression |
| 4 | Bonaventure 2007 | SB-269970 | (2R)-1-[(3-hydroxyphenyl)sulfonyl]-2-[2-(4-methyl-1-piperidinyl)ethyl]-pyrrolidine | vehicle | TST | depression |
| 5 | Bonaventure 2012 | JNJ-18038683 | (3-(4-chlorophenyl)-1,4,5,6,7,8- hexahydro-1-(phenylmethyl)pyrazolo[3,4-d]azepine 2-hydroxy-1,2,3-propanetricarboxylate) | vehicle | TST | depression |
| 6 | Canale 2015 | Compound 32 | (S)-2-(5-(4-([1,1'-Biphenyl]-2-yl)piperazin-1-yl)pentanoyl)-1,2,3,4-tetrahydroisoquinoline-3-carboxamide | vehicle | FST | depression |
| **No.** | **Author / Year** | **Compound Name** | **Chemical Name** | **Groups and/or Comparator** | **Tests** | **Model** |
| 7 | Canale 2016a | Compound 17 | 1-Methyl-N-(1-{[2-(t-butyl-2-yl)phenoxy]ethyl}piperidin-4-yl)-N-methyl-1H-pyrazole-sulfonamide | vehicle | FST | depression |
| 7 | Canale 2016a | Compound 20 | 1-Methyl-N-{1-[2-(biphenyl-2-yloxy)ethyl]piperidin-4-yl}-N-methyl-1H-pyrazole-4-sulfonamide | vehicle | FST | depression |
| 7 | Canale 2016a | Compound 31 | (1-methyl-N-{1-[2-(2-(t-butyl)phenoxy)ethyl]piperidin-4-yl}-N-cyclopropylmethyl-1H-pyrazole-4-sulfonamide) | vehicle | FST | depression |
| 7 | Canale 2016a | Compound 33 | 1-Methyl-N-{1-[2-(biphenyl-2-yloxy)ethyl]piperidin-4-yl}-N-cyclopropylmethyl-1H-pyrazole-4-sulfonamide | vehicle | FST | depression |
| 7 | Canale 2016a | Compound 31 | (1-methyl-N-{1-[2-(2-(t-butyl)phenoxy)ethyl]piperidin-4-yl}-N-cyclopropylmethyl-1H-pyrazole-4-sulfonamide) | PCP (induced memory impairment) | NOR | cognition |
| 7 | Canale 2016a | Compound 33 | 1-Methyl-N-{1-[2-(biphenyl-2-yloxy)ethyl]piperidin-4-yl}-N-cyclopropylmethyl-1H-pyrazole-4-sulfonamide | PCP (induced memory impairment) | NOR | cognition |
| 8 | Canale 2016b | PZ-1417 (27) | 3-fluoro-N-{1-[2-(2-cyclopentylphenoxy)ethyl]piperidin-4-yl}-benzenesulfonamide; PZ-1417 | vehicle | FST | depression |
| 8 | Canale 2016b | PZ-1150 (35) | 4-fluoro-N-(1-{2-[(propan-2-yl)phenoxy]ethyl}-8-azabicyclo[3.2.1]octan-3-yl)-benzenesulfonamide | vehicle | FST | depression |
| 8 | Canale 2016b | PZ-1417 (27) | 3-fluoro-N-{1-[2-(2-cyclopentylphenoxy)ethyl]piperidin-4-yl}-benzenesulfonamide; PZ-1417 | vehicle | FPT | anxiety |
| 8 | Canale 2016b | PZ-1150 (35) | 4-fluoro-N-(1-{2-[(propan-2-yl)phenoxy]ethyl}-8-azabicyclo[3.2.1]octan-3-yl)-benzenesulfonamide | vehicle | FPT | anxiety |
| 8 | Canale 2016b | PZ-1417 (27) | 3-fluoro-N-{1-[2-(2-cyclopentylphenoxy)ethyl]piperidin-4-yl}-benzenesulfonamide; PZ-1417 | vehicle | TST | depression |
| 8 | Canale 2016b | PZ-1150 (35) | 4-fluoro-N-(1-{2-[(propan-2-yl)phenoxy]ethyl}-8-azabicyclo[3.2.1]octan-3-yl)-benzenesulfonamide | vehicle | TST | depression |
| 9 | Canale 2017 | Compound 20 | 4-Fluoro-N-(1-{3-[(2-isopropylphenoxy)]propyl}piperidin-4-yl)benzenesulfonamide | vehicle | FST | depression |
| 9 | Canale 2017 | Compound 25 | 3-chloro-N-{1-[3-(1,1-biphenyl-2- yloxy)2-hydroxypropyl]piperidin-4-yl}benzenesulfonamide | vehicle | FST | depression |
| 9 | Canale 2017 | Compound 20 | 4-Fluoro-N-(1-{3-[(2-isopropylphenoxy)]propyl}piperidin-4-yl)benzenesulfonamide | vehicle | TST | depression |
| **No.** | **Author / Year** | **Compound Name** | **Chemical Name** | **Groups and/or Comparator** | **Tests** | **Model** |
| 9 | Canale 2017 | Compound 25 | 3-chloro-N-{1-[3-(1,1-biphenyl-2- yloxy)2-hydroxypropyl]piperidin-4-yl}benzenesulfonamide | vehicle | TST | depression |
| 9 | Canale 2017 | Compound 20 | 4-Fluoro-N-(1-{3-[(2-isopropylphenoxy)]propyl}piperidin-4-yl)benzenesulfonamide | vehicle | NOR | cognition |
| 9 | Canale 2017 | Compound 25 | 3-chloro-N-{1-[3-(1,1-biphenyl-2- yloxy)2-hydroxypropyl]piperidin-4-yl}benzenesulfonamide | vehicle | NOR | cognition |
| 10 | Cates 2013 | Lurasidone | (3aR,4S,7R,7aS)-2-{(1R,2R)-2-[4-(1,2-benzisothiazol-3-yl)piperazin-1-ylmethyl] cyclohexylmethyl}hexahydro-4,7-methano-2H-isoindole-1,3-dione | 5-HT7+/+ (WT) mice vs 5-HT7-/- (KO) mice; vehicle | TST | depression |
| 10 | Cates 2013 | Lurasidone | (3aR,4S,7R,7aS)-2-{(1R,2R)-2-[4-(1,2-benzisothiazol-3-yl)piperazin-1-ylmethyl] cyclohexylmethyl}hexahydro-4,7-methano-2H-isoindole-1,3-dione | 5-HT7+/+ (WT) mice vs 5-HT7-/- (KO) mice; vehicle | FST | depression |
| 10 | Cates 2013 | Lurasidone | (3aR,4S,7R,7aS)-2-{(1R,2R)-2-[4-(1,2-benzisothiazol-3-yl)piperazin-1-ylmethyl] cyclohexylmethyl}hexahydro-4,7-methano-2H-isoindole-1,3-dione | vehicle | OSST | depression |
| 10 | Cates 2013 | Lurasidone | (3aR,4S,7R,7aS)-2-{(1R,2R)-2-[4-(1,2-benzisothiazol-3-yl)piperazin-1-ylmethyl] cyclohexylmethyl}hexahydro-4,7-methano-2H-isoindole-1,3-dione | 5-HT7+/+ (WT) mice vs 5-HT7-/- (KO) mice; vehicle | D/L | anxiety |
| 10 | Cates 2013 | Lurasidone | (3aR,4S,7R,7aS)-2-{(1R,2R)-2-[4-(1,2-benzisothiazol-3-yl)piperazin-1-ylmethyl] cyclohexylmethyl}hexahydro-4,7-methano-2H-isoindole-1,3-dione | 5-HT7+/+ (WT) mice vs 5-HT7-/- (KO) mice; vehicle | MB | anxiety |
| 11 | Chlon-Rzepa 2013 | Compound 21 | 7-Benzyl-8-((4-(4-(3-chlorophenyl)piperazin-1- yl)butyl)amino)-1,3-dimethyl-1H-purine2,6(3H,7H)-dione hydrochloride | vehicle | FST | depression |
| 11 | Chlon-Rzepa 2013 | Compound 42 | 8-(3-(4-(4-Fluorophenyl)piperazin-1-yl)propoxy)-1,3-dimethyl-7-(3-phenylpropyl)- 1H-purine-2,6(3H,7H)-dione hydrochloride | vehicle | FST | depression |
| **No.** | **Author / Year** | **Compound Name** | **Chemical Name** | **Groups and/or Comparator** | **Tests** | **Model** |
| 11 | Chlon-Rzepa 2013 | Compound 21 | 7-Benzyl-8-((4-(4-(3-chlorophenyl)piperazin-1- yl)butyl)amino)-1,3-dimethyl-1H-purine2,6(3H,7H)-dione hydrochloride | vehicle | FPT | anxiety |
| 11 | Chlon-Rzepa 2013 | Compound 42 | 8-(3-(4-(4-Fluorophenyl)piperazin-1-yl)propoxy)-1,3-dimethyl-7-(3-phenylpropyl)- 1H-purine-2,6(3H,7H)-dione hydrochloride | vehicle | FPT | anxiety |
| 11 | Chlon-Rzepa 2013 | Compound 21 | 7-Benzyl-8-((4-(4-(3-chlorophenyl)piperazin-1- yl)butyl)amino)-1,3-dimethyl-1H-purine2,6(3H,7H)-dione hydrochloride | vehicle | random locomotor activity |  |
| 11 | Chlon-Rzepa 2013 | Compound 42 | 8-(3-(4-(4-Fluorophenyl)piperazin-1-yl)propoxy)-1,3-dimethyl-7-(3-phenylpropyl)- 1H-purine-2,6(3H,7H)-dione hydrochloride | vehicle | random locomotor activity |  |
| 12 | Delcourte 2017 | Asenapine | (3aRS,12bRS)-rel-5-Chloro-2,3,3a,12b-tetrahydro- 2-methyl-1H-dibenz[2,3:6,7]oxepino[4,5-c]pyrrole | sleep deprevation model of mania | locomotor activity | mania |
| 12 | Delcourte 2017 | Asenapine | (3aRS,12bRS)-rel-5-Chloro-2,3,3a,12b-tetrahydro- 2-methyl-1H-dibenz[2,3:6,7]oxepino[4,5-c]pyrrole | sleep deprevation model of mania | locomotor activity | mania |
| 12 | Delcourte 2017 | Asenapine | (3aRS,12bRS)-rel-5-Chloro-2,3,3a,12b-tetrahydro- 2-methyl-1H-dibenz[2,3:6,7]oxepino[4,5-c]pyrrole | vehicle | FST | depression |
| 12 | Delcourte 2017 | Asenapine | (3aRS,12bRS)-rel-5-Chloro-2,3,3a,12b-tetrahydro- 2-methyl-1H-dibenz[2,3:6,7]oxepino[4,5-c]pyrrole | vehicle - (ATCH model treatment resistant depression) | FST | treatment resistant depression |
| 13 | Gu 2017 | Compound 8j | 3-(4-(3,4-dichlorophenethyl)piperazin-1-yl)benzo[d]isothiazole hydrochloride | vehicle | FST | depression |
| 14 | Gu 2018 | Compound 21n | 5-Fluoro-3-(3-(4-(5-fluoro-[1,1′-biphenyl]-2-yl)piperazin-1-yl)propyl)-1H-indole hydrochloride | vehicle | FST | depression |
| 14 | Gu 2018 | Compound 21n | 5-Fluoro-3-(3-(4-(5-fluoro-[1,1′-biphenyl]-2-yl)piperazin-1-yl)propyl)-1H-indole hydrochloride | vehicle | TST | depression |
| 15 | Gu 2019 | Compound 19a | 5-fluoro-3-(3-(4-(5-fluoro-[1,1'-biphenyl]-2-yl)piperidin-1-yl)propyl)-1H-indole | vehicle | FST | depression |
| **No.** | **Author / Year** | **Compound Name** | **Chemical Name** | **Groups and/or Comparator** | **Tests** | **Model** |
| 15 | Gu 2019 | Compound 19a | 5-fluoro-3-(3-(4-(5-fluoro-[1,1'-biphenyl]-2-yl)piperidin-1-yl)propyl)-1H-indole | vehicle | TST | depression |
| 16 | Guilloux 2013 | Vortioxitine | 1-[2-(2,4-dimethyl-phenylsulfanyl)-phenyl]-piperazine | vehicle | OFT | anxiety |
| 16 | Guilloux 2013 | Vortioxitine | 1-[2-(2,4-dimethyl-phenylsulfanyl)-phenyl]-piperazine | vehicle | FST | depression |
| 17 | Guscott 2005 | SB-258719 (and KO mice) | (1R)-3,N-dimethyl-N-[1-methyl-3-(4-methylpiperidin-1-yl)propyl]benzenesulfonamide | vehicle | FST | depression |
| 18 | Hedlund 2005 | SB-269970 (and 5HT7 KO) | (2R)-1-[(3-hydroxyphenyl)sulfonyl]-2-[2-(4-methyl-1-piperidinyl)ethyl]-pyrrolidine | 5-HT7+/+ (WT) mice vs 5-HT7-/- (KO) mice; vehicle | FST | depression |
| 18 | Hedlund 2005 | SB-269970 (and 5HT7 KO) | (2R)-1-[(3-hydroxyphenyl)sulfonyl]-2-[2-(4-methyl-1-piperidinyl)ethyl]-pyrrolidine | 5-HT7+/+ (WT) mice vs 5-HT7-/- (KO) mice; vehicle | FST | depression |
| 18 | Hedlund 2005 | SB-269970 (and 5HT7 KO) | (2R)-1-[(3-hydroxyphenyl)sulfonyl]-2-[2-(4-methyl-1-piperidinyl)ethyl]-pyrrolidine | 5-HT7+/+ (WT) mice vs 5-HT7-/- (KO) mice; vehicle | TST | depression |
| 18 | Hedlund 2005 | SB-269970 (and 5HT7 KO) | (2R)-1-[(3-hydroxyphenyl)sulfonyl]-2-[2-(4-methyl-1-piperidinyl)ethyl]-pyrrolidine | 5-HT7+/+ (WT) mice vs 5-HT7-/- (KO) mice; vehicle | TST | depression |
| 19 | Hedlund 2007 | SB-269970 (and 5HT7 KO) | (2R)-1-[(3-hydroxyphenyl)sulfonyl]-2-[2-(4-methyl-1-piperidinyl)ethyl]-pyrrolidine | vehicle - 5HT7 KO and WT mice | MB | OCD/repetative behaviours/anxiety |
| 19 | Hedlund 2007 | SB-269970 (and 5HT7 KO) | (2R)-1-[(3-hydroxyphenyl)sulfonyl]-2-[2-(4-methyl-1-piperidinyl)ethyl]-pyrrolidine | vehicle - 5HT7 KO and WT mice | MB | OCD/repetative behaviours/anxiety |
| 20 | Jankowska 2020 | Compound 22 | N-(4-isopropylphenyl)-5-(4-(2-methoxyphenyl)piperazin-1-yl)pentanamide hydrochloride | MK-801 (induced memory impairment) | NOR | cognition |
| 20 | Jankowska 2020 | Compound 22 | N-(4-isopropylphenyl)-5-(4-(2-methoxyphenyl)piperazin-1-yl)pentanamide hydrochloride | vehicle | FST | depression |
| 21 | Kim 2014 | Compound 1-8 | N-((2′-Chlorobiphenyl-2-yl)methyl)-5-(4-(2-methoxyphenyl)piperazin-1-yl)pentanamide | vehicle | FST | depression |
| 22 | Kim 2016 | Compound 28 | 1-(9H-carbazol-9-yl)-6-(4-(2-methoxyphenyl)piperazin-1-yl)hexan-1-one hydrochloride | vehicle | FST | depression |
| **No.** | **Author / Year** | **Compound Name** | **Chemical Name** | **Groups and/or Comparator** | **Tests** | **Model** |
| 23 | Kolaczkowski 2014 | ADN-1184 | 1-(1-adamantyl)-N-1-naphthyl-4-nitro-1H-pyrazole-3-carboxamide | vehicle | FST | depression |
| 24 | Kucwaj-Brysz 2018 | Compound 5 | 5-Phenyl-3-(3-(4-(2-methoxyphenyl)piperazin-1-yl)-2-hydroxypropyl)-5-methylimidazolidine-2,4-dione hydrochloride | vehicle | FST | depression |
| 24 | Kucwaj-Brysz 2018 | Compound 6 | 5-Phenyl-3-(3-(4-(2-cyanophenyl)piperazin-1-yl)-2-hydroxypropyl)-5-methylimidazolidine-2,4-dione hydrochloride | vehicle | FST | depression |
| 24 | Kucwaj-Brysz 2018 | Compound 7 | 5-(4-Bromophenyl)-3-(3-(4-(2-methoxyphenyl)piperazin-1-yl)-2-hydroxypropyl)-5-methylimidazolidine-2,4-dione hydrochloride | vehicle | FST | depression |
| 24 | Kucwaj-Brysz 2018 | Compound 8 | 5-(4-Bromophenyl)-3-(3-(4-(2-cyanophenyl)piperazin-1-yl)-2-hydroxypropyl)-5-methylimidazolidine-2,4-dione hydrochloride | vehicle | FST | depression |
| 24 | Kucwaj-Brysz 2018 | Compound 5 | 5-Phenyl-3-(3-(4-(2-methoxyphenyl)piperazin-1-yl)-2-hydroxypropyl)-5-methylimidazolidine-2,4-dione hydrochloride | vehicle | FPT | anxiety |
| 24 | Kucwaj-Brysz 2018 | Compound 6 | 5-Phenyl-3-(3-(4-(2-cyanophenyl)piperazin-1-yl)-2-hydroxypropyl)-5-methylimidazolidine-2,4-dione hydrochloride | vehicle | FPT | anxiety |
| 24 | Kucwaj-Brysz 2018 | Compound 7 | 5-(4-Bromophenyl)-3-(3-(4-(2-methoxyphenyl)piperazin-1-yl)-2-hydroxypropyl)-5-methylimidazolidine-2,4-dione hydrochloride | vehicle | FPT | anxiety |
| 24 | Kucwaj-Brysz 2018 | Compound 8 | 5-(4-Bromophenyl)-3-(3-(4-(2-cyanophenyl)piperazin-1-yl)-2-hydroxypropyl)-5-methylimidazolidine-2,4-dione hydrochloride | vehicle | FPT | anxiety |
| 25 | Latacz 2018 | MF-8 | (5-(4-fluorophenyl)-3-(2-hydroxy-3-(4-(2-methoxyphenyl)piperazin-1-yl)propyl)-5-methylhydantoin) | vehicle | FST - mice | depression |
| 25 | Latacz 2018 | MF-8 | (5-(4-fluorophenyl)-3-(2-hydroxy-3-(4-(2-methoxyphenyl)piperazin-1-yl)propyl)-5-methylhydantoin) | vehicle | FST - rats | depression |
| **No.** | **Author / Year** | **Compound Name** | **Chemical Name** | **Groups and/or Comparator** | **Tests** | **Model** |
| 26 | Lax 2018 | DUQ0002-I |  | vehicle | D/L | anxiety |
| 26 | Lax 2018 | DUQ0002-I |  | vehicle | EOM | anxiety |
| 26 | Lax 2018 | DUQ0002-I |  | vehicle | TST | depresion |
| 26 | Lax 2018 | DUQ0002-I |  | vehicle | FST | depression |
| 27 | Li 2013 | Vortioxitine | 1-[2-(2,4-dimethyl-phenylsulfanyl)-phenyl]-piperazine | progesterone withdrawl induced depression - vehicle | FST | depression (hormonal) |
| 27 | Li 2013 | SB-269970 | (2R)-1-[(3-hydroxyphenyl)sulfonyl]-2-[2-(4-methyl-1-piperidinyl)ethyl]-pyrrolidine | progesterone withdrawl induced depression - vehicle | FST | depression (hormonal) |
| 27 | Li 2013 | AS-19 |  | progesterone withdrawl induced depression - vehicle | FST | depression (hormonal) |
| 28 | Maxwell 2019 | DR-4004 |  | vehicle | EMT | anxiety |
| 28 | Maxwell 2019 | SB-269970 | (2R)-1-[(3-hydroxyphenyl)sulfonyl]-2-[2-(4-methyl-1-piperidinyl)ethyl]-pyrrolidine | vehicle | EMT | anxiety |
| 28 | Maxwell 2019 | DR-4004 |  | vehicle | TST | depression |
| 28 | Maxwell 2019 | SB-269970 | (2R)-1-[(3-hydroxyphenyl)sulfonyl]-2-[2-(4-methyl-1-piperidinyl)ethyl]-pyrrolidine | vehicle | TST | depression |
| 29 | Medina 2014 | Compound 6 | 1-[(3E)-6-(3,4-Dihydroisoquinolin-2(1H)-yl)hex-3-en-1-yl]-1,3-dihydro-2H-indol-2-one | vehicle | TST | depression |
| 29 | Medina 2014 | Compound 6 | 1-[(3E)-6-(3,4-Dihydroisoquinolin-2(1H)-yl)hex-3-en-1-yl]-1,3-dihydro-2H-indol-2-one | vehicle | FST | depression |
| 30 | Mnie-Filali 2011 | SB-269970 | (2R)-1-[(3-hydroxyphenyl)sulfonyl]-2-[2-(4-methyl-1-piperidinyl)ethyl]-pyrrolidine | vehicle / vehicle + fluoxetine | OFT | anxiety |
| 31 | Mork 2012 | Lu AA21004 | 1-[2-(2,4-Dimethylphenylsulfanyl)phenyl]piperazine | Flinders Sensitive Line (FSL) rat and Flinders Resistant Line (FRL) rat, have been selectively bred for high and low sensitivity to cholinergic agonism, respectively / groups compared to vehicle | FST | depression |
| 31 | Mork 2012 | Lu AA21004 | 1-[2-(2,4-Dimethylphenylsulfanyl)phenyl]piperazine | Flinders Sensitive Line (FSL) rat and Flinders Resistant Line (FRL) rat / vehicle | CFIV | anxiety |
| **No.** | **Author / Year** | **Compound Name** | **Chemical Name** | **Groups and/or Comparator** | **Tests** | **Model** |
| 32 | Partyka 2017 | Compound 16 | 4-({4-(2-[4-(3-Chlorophenyl)piperazin-1-yl]ethyl)piperidin-1-yl}sulfonyl)isoquinoline | vehicle | FST | depression |
| 33 | Partyka 2019 | PZ-1433 (compound 20 in Canale 2017) | (4-Fluoro-N-(1-{3-[(2-isopropylphenoxy)]propyl}piperidin-4 yl)benzenesulfonamide) | vehicle | FST | depression |
| 33 | Partyka 2019 | ADN-1184 | 1-(1-adamantyl)-N-1-naphthyl-4-nitro-1H-pyrazole-3-carboxamide | vehicle | FST | depression |
| 33 | Partyka 2019 | PZ-1433 (compound 20 in Canale 2017) | (4-Fluoro-N-(1-{3-[(2-isopropylphenoxy)]propyl}piperidin-4 yl)benzenesulfonamide) | 10mg citalopram / 10mg buproprion | FST | depression |
| 33 | Partyka 2019 | ADN-1184 | 1-(1-adamantyl)-N-1-naphthyl-4-nitro-1H-pyrazole-3-carboxamide | 10mg citalopram / 10mg buproprion | FST | depression |
| 34 | Pytka 2015 | HBK-14 | 1-{2-[2-(2,6-dimethlphenoxy)ethoxy]ethyl}-4-(2-methoxyphenyl)piperazynine hydrochloride | vehicle | FST (mice) | depression |
| 34 | Pytka 2015 | HBK-15 | 2-[2-(2-chloro-6-methylphenoxy)ethoxy]ethyl-4-(2- methoxyphenyl)piperazynine dihydrochloride | vehicle | FST (mice) | depression |
| 34 | Pytka 2015 | HBK-14 | 1-{2-[2-(2,6-dimethlphenoxy)ethoxy]ethyl}-4-(2-methoxyphenyl)piperazynine hydrochloride | vehicle | FST (rats) | depression |
| 34 | Pytka 2015 | HBK-15 | 2-[2-(2-chloro-6-methylphenoxy)ethoxy]ethyl-4-(2- methoxyphenyl)piperazynine dihydrochloride | vehicle | FST (rats) | depression |
| 34 | Pytka 2015 | HBK-14 | 1-{2-[2-(2,6-dimethlphenoxy)ethoxy]ethyl}-4-(2-methoxyphenyl)piperazynine hydrochloride | vehicle | EMT | anxiety |
| 34 | Pytka 2015 | HBK-15 | 2-[2-(2-chloro-6-methylphenoxy)ethoxy]ethyl-4-(2- methoxyphenyl)piperazynine dihydrochloride | vehicle | EMT | anxiety |
| 34 | Pytka 2015 | HBK-14 | 1-{2-[2-(2,6-dimethlphenoxy)ethoxy]ethyl}-4-(2-methoxyphenyl)piperazynine hydrochloride | vehicle | FPT | anxiety |
| 34 | Pytka 2015 | HBK-15 | 2-[2-(2-chloro-6-methylphenoxy)ethoxy]ethyl-4-(2- methoxyphenyl)piperazynine dihydrochloride | vehicle | FPT | anxiety |
| **No.** | **Author / Year** | **Compound Name** | **Chemical Name** | **Groups and/or Comparator** | **Tests** | **Model** |
| 35 | Pytka 2017a | HBK-15 | 2-[2-(2-chloro-6-methylphenoxy)ethoxy]ethyl-4-(2- methoxyphenyl)piperazynine dihydrochloride | vehicle | FST | depression |
| 35 | Pytka 2017a | HBK-14 | 1-{2-[2-(2,6-dimethlphenoxy)ethoxy]ethyl}-4-(2-methoxyphenyl)piperazynine hydrochloride | vehicle | STPA | cognition |
| 35 | Pytka 2017a | HBK-15 | 2-[2-(2-chloro-6-methylphenoxy)ethoxy]ethyl-4-(2- methoxyphenyl)piperazynine dihydrochloride | vehicle | STPA | cognition |
| 36 | Pytka 2017b | HBK-15 | 2-[2-(2-chloro-6-methylphenoxy)ethoxy]ethyl-4-(2- methoxyphenyl)piperazynine dihydrochloride | vehicle in chronically stressed mice | FST | depression + chronic stress |
| 36 | Pytka 2017b | HBK-15 | 2-[2-(2-chloro-6-methylphenoxy)ethoxy]ethyl-4-(2- methoxyphenyl)piperazynine dihydrochloride | vehicle in chronically stressed mice | SCT | depression + chronic stress |
| 36 | Pytka 2017b | HBK-15 | 2-[2-(2-chloro-6-methylphenoxy)ethoxy]ethyl-4-(2- methoxyphenyl)piperazynine dihydrochloride | vehicle in chronically stressed mice | EMT | anxiety + chronic stress |
| 37 | Pytka 2018 | HBK-14 | 1-[(2,6-Dimethylphenoxy)ethoxyethyl]-4-(2-methoxyphenyl)piperazine hydrochloride | vehicle in stressed mice (treated corticosterone) | FST | depression + stress |
| 37 | Pytka 2018 | HBK-15 | 1-[(2-chloro-6-methylphenoxy)ethoxyethyl]-4-(2-methoxyphenyl)piperazine hydrochloride | vehicle in stressed mice (treated corticosterone) | FST | depression + stress |
| 37 | Pytka 2018 | HBK-14 | 1-[(2,6-Dimethylphenoxy)ethoxyethyl]-4-(2-methoxyphenyl)piperazine hydrochloride | vehicle in stressed mice (treated corticosterone) | SCT | depression + stress |
| 37 | Pytka 2018 | HBK-15 | 1-[(2-chloro-6-methylphenoxy)ethoxyethyl]-4-(2-methoxyphenyl)piperazine hydrochloride | vehicle in stressed mice (treated corticosterone) | SCT | depression + stress |
| 37 | Pytka 2018 | HBK-14 | 1-[(2,6-Dimethylphenoxy)ethoxyethyl]-4-(2-methoxyphenyl)piperazine hydrochloride | vehicle in stressed mice (treated corticosterone) | EMT | anxiety + stress |
| 37 | Pytka 2018 | HBK-15 | 1-[(2-chloro-6-methylphenoxy)ethoxyethyl]-4-(2-methoxyphenyl)piperazine hydrochloride | vehicle in stressed mice (treated corticosterone) | EMT | anxiety + stress |
| 38 | Stroth 2015 | SB-269970 | (2R)-1-[(3-hydroxyphenyl)sulfonyl]-2-[2-(4-methyl-1-piperidinyl)ethyl]-pyrrolidine | S100B-overexpressing transgenic mice (basal immobility 32% higher than WT mice) + vehicle | FST | depression |
| 39 | Volk 2008 | Compound 9e' | 3-{4-[4-(4-Chlorophenyl)-piperazin-1-yl]-butyl}-3-ethyl-6-fluoro-1,3-dihydro-2H-indol-2-one | vehicle | vCDT | anxiety |
| 39 | Volk 2008 | Compound 12d | 3-{4-[4-(4-Chloro-phenyl)-piperazin-1-yl]-butyl}-1,3-dihydro-2H-indol-2-one | vehicle | vCDT | anxiety |
| **No.** | **Author / Year** | **Compound Name** | **Chemical Name** | **Groups and/or Comparator** | **Tests** | **Model** |
| 39 | Volk 2008 | Compound12e | 5-Fluoro-3-[4-(4-phenyl-piperazin-1-il)-butyl]-1,3-dihydro-2H-indol-2-one | vehicle | vCDT | anxiety |
| 39 | Volk 2008 | Compound 9e' | 3-{4-[4-(4-Chlorophenyl)-piperazin-1-yl]-butyl}-3-ethyl-6-fluoro-1,3-dihydro-2H-indol-2-one | vehicle | D/L | anxiety |
| 39 | Volk 2008 | Compound 12d | 3-{4-[4-(4-Chloro-phenyl)-piperazin-1-yl]-butyl}-1,3-dihydro-2H-indol-2-one | vehicle | D/L | anxiety |
| 39 | Volk 2008 | Compound12e | 5-Fluoro-3-[4-(4-phenyl-piperazin-1-il)-butyl]-1,3-dihydro-2H-indol-2-one | vehicle | D/L | anxiety |
| 40 | Volk 2011 | Compound 1a | 3-{4-[4-(4-chlorophenyl)piperazin-1-yl]butyl}-3-ethyl-6-fluoro-1,3-dihydro-2H-indol-2-one | vehicle | vCDT | anxiety |
| 40 | Volk 2011 | Compound 2a |  | vehicle | vCDT | anxiety |
| 40 | Volk 2011 | Compound 1a | 3-{4-[4-(4-chlorophenyl)piperazin-1-yl]butyl}-3-ethyl-6-fluoro-1,3-dihydro-2H-indol-2-one | vehicle | D/L | anxiety |
| 40 | Volk 2011 | Compound 2a |  | vehicle | D/L | anxiety |
| 40 | Volk 2011 | Compound 1a | 3-{4-[4-(4-chlorophenyl)piperazin-1-yl]butyl}-3-ethyl-6-fluoro-1,3-dihydro-2H-indol-2-one | vehicle | FST | depression |
| 40 | Volk 2011 | Compound 2a |  | vehicle | FST | depression |
| 41 | Wang 2019 | Compound 7a | 5-fluoro-3-(1-(2-((5-fluoro-[1,1'-biphenyl]-2-yl)oxy)ethyl)piperidin-4-yl)-1H-indole | vehicle | FST | depression |
| 41 | Wang 2019 | Compound 15g | 3-(1-(2-((3',5-difluoro-[1,1'-biphenyl]-2-yl)oxy)ethyl)piperidin-4-yl)-5-fluoro-1H-indole | vehicle | FST | depression |
| 41 | Wang 2019 | Compound 7a | 5-fluoro-3-(1-(2-((5-fluoro-[1,1'-biphenyl]-2-yl)oxy)ethyl)piperidin-4-yl)-1H-indole | vehicle | TST | depression |
| 41 | Wang 2019 | Compound 15g | 3-(1-(2-((3',5-difluoro-[1,1'-biphenyl]-2-yl)oxy)ethyl)piperidin-4-yl)-5-fluoro-1H-indole | vehicle | TST | depression |
| 42 | Waszkielewicz 2015 | Compound 2 | 1-[(2,5-dimethylphenoxy)propyl]-4-(2-methoxyphenyl)piperazine hydrochloride | vehicle | TST | depression |
| 42 | Waszkielewicz 2015 | Compound 3 | 1-[(2,3,5-trimethylphenoxy)propyl]-4-(2-methoxyphenyl)piperazine hydrochloride | vehicle | TST | depression |
| 42 | Waszkielewicz 2015 | Compound 6 | 1-[(2-chloro-6-methylphenoxy)ethoxyethyl]-4-(2-methoxyphenyl)piperazine hydrochloride | vehicle | TST | depression |
| **No.** | **Author / Year** | **Compound Name** | **Chemical Name** | **Groups and/or Comparator** | **Tests** | **Model** |
| 43 | Wesolowska 2006a | SB-269970 | (2R)-1-[(3-hydroxyphenyl)sulfonyl]-2-[2-(4-methyl-1-piperidinyl)ethyl]-pyrrolidine | vehicle | vCDT | anxiety |
| 43 | Wesolowska 2006a | SB-269970 | (2R)-1-[(3-hydroxyphenyl)sulfonyl]-2-[2-(4-methyl-1-piperidinyl)ethyl]-pyrrolidine | vehicle | FST | depression |
| 44 | Wesolowska 2006b | SB-269970 | (2R)-1-[(3-hydroxyphenyl)sulfonyl]-2-[2-(4-methyl-1-piperidinyl)ethyl]-pyrrolidine | vehicle | vCDT | anxiety |
| 44 | Wesolowska 2006b | SB-269970 | (2R)-1-[(3-hydroxyphenyl)sulfonyl]-2-[2-(4-methyl-1-piperidinyl)ethyl]-pyrrolidine | vehicle | EMT | anxiety |
| 44 | Wesolowska 2006b | SB-269970 | (2R)-1-[(3-hydroxyphenyl)sulfonyl]-2-[2-(4-methyl-1-piperidinyl)ethyl]-pyrrolidine | vehicle | FPT | anxiety |
| 44 | Wesolowska 2006b | SB-269970 | (2R)-1-[(3-hydroxyphenyl)sulfonyl]-2-[2-(4-methyl-1-piperidinyl)ethyl]-pyrrolidine | vehicle | FST | depression |
| 44 | Wesolowska 2006b | SB-269970 | (2R)-1-[(3-hydroxyphenyl)sulfonyl]-2-[2-(4-methyl-1-piperidinyl)ethyl]-pyrrolidine | vehicle | TST | depresion |
| 45 | Wesolowska 2007 | SB-269970 | (2R)-1-[(3-hydroxyphenyl)sulfonyl]-2-[2-(4-methyl-1-piperidinyl)ethyl]-pyrrolidine | low dose antidepressants | FST | depression |
| 46 | Wrobel 2019 | MW005 | 1-{4-[4-(5-fluoro-1H-indol-3-yl)piperidin-1-yl]butyl}-3-(1H-indol-3-yl)pyrrolidine-2,5-dione | vehicle | FST | depression |
| 46 | Wrobel 2019 | compound 4A | 1-{4-[4-(1H-indol-3-yl)piperidin-1-yl]butyl}-3-(5-methoxy-1H-indol-3-yl)pyrrolidine-2,5-dione | vehicle | FST | depression |
| 46 | Wrobel 2019 | compound 4J | 1-{4-[4-(1H-indol-3-yl)piperidin-1-yl]butyl}-3-(5-fluoro-1H-indol-3-yl)pyrrolidine-2,5-dione | vehicle | FST | depression |
| 47 | Zagorska 2015 | Compound 8 | 7-Phenyl-8-[4-(N4-2′-hydroxyphenyl)-piperazin-N1-yl-butyl]-1,3-dimethyl-(1H,8H)-imidazo[2,1-f]purine-2,4-dione | vehicle | FST | depression |
| 47 | Zagorska 2015 | Compound 9 | 7-Phenyl-8-[5-(N4-2′-hydroxyphenyl)-piperazin-N1-yl-pentyl]-1,3-dimethyl-(1H,8H)-imidazo[2,1-f]purine-2,4-dione | vehicle | FST | depression |
| 47 | Zagorska 2015 | Compound 8 | 7-Phenyl-8-[4-(N4-2′-hydroxyphenyl)-piperazin-N1-yl-butyl]-1,3-dimethyl-(1H,8H)-imidazo[2,1-f]purine-2,4-dione | vehicle | FPT | anxiety |
| **No.** | **Author / Year** | **Compound Name** | **Chemical Name** | **Groups and/or Comparator** | **Tests** | **Model** |
| 47 | Zagorska 2015 | Compound 9 | 7-Phenyl-8-[5-(N4-2′-hydroxyphenyl)-piperazin-N1-yl-pentyl]-1,3-dimethyl-(1H,8H)-imidazo[2,1-f]purine-2,4-dione | vehicle | FPT | anxiety |
| 48 | Zagorska 2016 | Compound 9 | 8-(5-(4-(2-fluorophenyl)piperazin-1-yl)pentyl)-1,3,7-trimethyl-1H-imidazo[2,1-f]purine-2,4(3H,8H)-dione | vehicle | FST | depression |
| 48 | Zagorska 2016 | Compound 9 | 8-(5-(4-(2-fluorophenyl)piperazin-1-yl)pentyl)-1,3,7-trimethyl-1H-imidazo[2,1-f]purine-2,4(3H,8H)-dione | vehicle | FPT | anxiety |
| 49 | Zajdel 2011 | Compound 54 | (N-Ethyl-N-[4-(1,2,3,4,4a,5,6,7,8,8a-decahydroisoquinolin-2-yl)butyl]-8-quinolinesulfonamide) | vehicle | FST | depression |
| 50 | Zajdel 2012 | Compound 36 | (4-(4-{2-[4-(4-chloro-phenyl)-piperazin-1-yl]-ethyl}-piperidine-1-sulfonyl)-isoquinoline) | vehicle | FST | depression |
| 50 | Zajdel 2012 | Compound 36 | (4-(4-{2-[4-(4-chloro-phenyl)-piperazin-1-yl]-ethyl}-piperidine-1-sulfonyl)-isoquinoline) | vehicle | EMT | anxiety |
| 51 | Zajdel 2013 | Compound 33 | (N-(3-(4-(2,3-dichlorophenyl)piperazin-1-yl)propyl)quinoline-7-sulfonamide) | vehicle | FST | depression |
| 51 | Zajdel 2013 | Compound 39 | (N-(4-(4-(2,3-dichlorophenyl)piperazin-1-yl)butyl)isoquinoline-3-sulfonamide) | vehicle | FST | depression |
| 52 | Zajdel 2015 | Compound 7 (PZ-766) | 4-fluoro-N-(1-{2-[(propan-2-yl)phenoxy]ethyl}piperidin-4-yl) benzenesulfonamide | vehicle | FST | depression |
| 52 | Zajdel 2015 | Compound 10 (PZ-1404) | (3-fluoro-N-(1-{2-[(propan-2-yl)phenoxy]ethyl}piperidin-4-yl)-benzenesulfonamide) | vehicle | FST | depression |
| 52 | Zajdel 2015 | Compound 7 (PZ-766) | 4-fluoro-N-(1-{2-[(propan-2-yl)phenoxy]ethyl}piperidin-4-yl) benzenesulfonamide | vehicle | FPT | anxiety |
| 52 | Zajdel 2015 | Compound 10 (PZ-1404) | (3-fluoro-N-(1-{2-[(propan-2-yl)phenoxy]ethyl}piperidin-4-yl)-benzenesulfonamide) | vehicle | FPT | anxiety |
| 52 | Zajdel 2015 | Compound 7 (PZ-766) | 4-fluoro-N-(1-{2-[(propan-2-yl)phenoxy]ethyl}piperidin-4-yl) benzenesulfonamide | PCP (induced memory impairment) | NOR | cognition |
| 52 | Zajdel 2015 | Compound 10 (PZ-1404) | (3-fluoro-N-(1-{2-[(propan-2-yl)phenoxy]ethyl}piperidin-4-yl)-benzenesulfonamide) | PCP (induced memory impairment) | NOR | cognition |

| **No.** | **Results** | **P-Value** | **Other notes** |
| --- | --- | --- | --- |
| 1 | Amisulpride decreased immobility time at 1mg/kg, but not 0.3,3, or 10mg/kg in WT mice; amisulpride did not decrease immobility time in KO mice (which have already decreased immobilty time compared to WT mice) | p<0.05 |  |
| 1 | Amisulpride decreased immobility time at 0.1mg/kg but not 0.3 or 1 mg/kg; amisulpride did not decrease immobility time in KO mice (which have already decreased immobilty time compared to WT mice) | p<0.01 |  |
| 2 | LP-211 administration (acutely, at a 0.25 mg/kg dose i.p.) increases locomotion and BWB exploration | p<0.05 |  |
| 2 | significantly at 0.25 mg/kg and as a tendency at 0.10 mg/kg dosages, mice spent much more time in the lit chamber than subjects of the vehicle group, revealing a decrease of anxiety-like behavior. | p<0.05 |  |
| 2 | animals administered with drug (at 0.25 mg/kg immediately before the retrieval) spent less time in the novel chamber, significantly so for the second 5-min partial interval | p<0.05 |  |
| 2 | mice treated with higher (0.83 and 2.5 mg/kg) doses spent more time in the white side in comparison with control mice | p<0.05 |  |
| 3 | KO mice displayed significant increase in latency to approach the shock probe | p<0.05 |  |
| 3 | KO mice had signifcantly lower latency to eat than WT mice | p<0.05 |  |
| 3 | no significant differences | NS |  |
| 3 | KO mice were signficantly less immobile | p<0.05 |  |
| 4 | 3mg/kg SB-269970 significantly decreased the immobility time compared with vehicle-treated mice | p<0.05 |  |
| 4 | 10mg/kg SB-269970 significantly decreased the immobility time compared with vehicle-treated mice | p<0.001 |  |
| 4 | 30mg/kg SB-269970 significantly decreased the immobility time compared with vehicle-treated mice | p<0.001 |  |
| 4 | 10mg/kg SB-269970 added to 1mg/kg citalopram alone reduced imobility time | p<0.001 |  |
| 5 | doses of 0.3, 0.5, and 1 mg/kg significantly decreased the immobility time | 0.3mg/kg - p<0.05; 0.5,1mg/kg - p<0.01 |  |
| 6 | 32 given in a dose of 10 mg/kg produced a distinct antidepressant-like effect in that test, significantly shortening the immobility time of mice by 24%; did not affect spontaneous locomotor activity | NR |  |
| **No.** | **Results** | **P-Value** | **Other notes** |
| 7 | 1.25mg/kg reduced immobility time compared to control | p<0.05 |  |
| 7 | 2.5mg/kg reduced immobility time compared to control | p<0.05 |  |
| 7 | 1.25mg/kg reduced immobility time compared to control | p<0.01 |  |
| 7 | 1.25mg/kg reduced immobility time compared to control | p<0.01 |  |
| 7 | found to dose-dependently (1 mg/kg, ip) ameliorate PCP-induced memory deficits in rats, with no influence on exploratory activity | p<0.001 |  |
| 7 | found to dose-dependently (1 mg/kg, ip) ameliorate PCP-induced memory deficits in rats, with no influence on exploratory activity | p<0.001 |  |
| 8 | signficantly reduced immobility time and was active over a wide range of doses (0.625–5 mg/kg) | p<0.05-p<0.001 |  |
| 8 | signficantly reduced immobility time at dose of 0.625 mg/kg | p<0.05 |  |
| 8 | exerted anxiolytic-like activity via increase in the number of shocks accepted by mice at dose of 1.25 and 2.5 mg/kg | p<0.05 |  |
| 8 | exerted anxiolytic-like activity via increase in the number of shocks accepted by mice at a dose of 0.625 mg/kg | p<0.05 |  |
| 8 | signficantly reduced immobility time at dose of 2.5 mg/kg | p<0.05 |  |
| 8 | did not significanlty reduce immobility time | NS |  |
| **No.** | **Results** | **P-Value** | **Other notes** |
| 9 | antidepressant-like activities at doses of 0.625–1.25 mg/kg, significantly reducing the duration of the immobility time by 19–53% | p<0.001 |  |
| 9 | antidepressant-like activities at doses of 0.625–2.5 mg/kg, significantly reducing the duration of the immobility time by 28–46% | p<0.05-p<0.001 |  |
| 9 | increased the average force of fights and decreased the immobility time when given at a dose of 2.5 mg/kg | force - p<0.05; immobility - p <0.01 |  |
| 9 | increased the average force of fights and decreased the immobility time when given at a dose of 2.5 mg/kg | both p<0.05 |  |
| 9 | acute administration reversed the natural forgetting impairment in rats (in 24 h interval) in a dose-dependent manner | p<0.001 |  |
| 9 | acute administration reversed the natural forgetting impairment in rats (in 24 h interval) in a dose-dependent manner | p<0.01-p<0.001 |  |
| 10 | reduced immobility in 5-HT7+/+ mice at the dose 0.3 mg/kg, but not at lower or higher doses (0.1, 1, 3mg/kg) | p<0.01 |  |
| 10 | reduced immobility in 5-HT7+/+ mice at the doses 0.3 and 1 mg/kg, but not at lower or higher doses (0.1 and 3mg/kg) | p<0.01 |  |
| 10 | 3 mg/kg, but not at 0.3 mg/kg, reversed the increased immobility back to levels close to baseline | p<0.05 |  |
| 10 | no significant differences | NS |  |
| 10 | no significant differences | NS |  |
| 11 | decreased immobility time at doses of 20 and 30 mg/kg compared to vehicle | 20mg/kg - p<0.05; 30mg/kg - p<0.001 |  |
| **No.** | **Results** | **P-Value** | **Other notes** |
| 11 | not significant | NS |  |
| 11 | increased punished crossings at 20mg/kg | p<0.05 |  |
| 11 | not significant | NS |  |
| 11 | administered at doses of 20 and 30 mg/ kg significantly decreased spontaneous locomotor activity of mice during 1-min and 4-min observation sessions | p<0.001 |  |
| 11 | produced a significant sedative effect during a 4-min observation period | p<0.001 |  |
| 12 | asenapine decreased hyperlocomotion of sleep deprived rats at 0.1mg/kg | p=0.52 |  |
| 12 | asenapine decreased hyperlocomotion of sleep deprived rats at 0.3mg/kg | p=0.001 |  |
| 12 | asenapine did not decrease immobility compared to control | NS |  |
| 12 | asenapine did not decrease immobility compared to control | NS |  |
| 13 | signficanlty reduced immobility time compared to vehicle at 20mg/kg and 40mg/kg | 20mg/kg - p<0.05; 40mg/kg - p<0.01 |  |
| 14 | signficanlty reduced immobility time compared to vehicle at 10, 20, and 40mg/kg | 10 & 20mg/kg - p<0.05; 40mg/kg - p<0.01 |  |
| 14 | signficanlty reduced immobility time compared to vehicle at 20 and 40mg/kg | 20mg/kg - p<0.05; 40mg/kg - p<0.01 |  |
| 15 | signficanlty reduced immobility time compared to vehicle at 20mg/kg and 40mg/kg | 20mg/kg - p<0.05; 40mg/kg - p<0.01 |  |
| 15 | signficanlty reduced immobility time compared to vehicle at 10, 20, and 40mg/kg | 10 mg/kg - p<0.05; 20 & 40mg/kg - p<0.01 |  |
| 16 | vortioxetine 2.5 and 5 mg/kg induced an anxiolytic-like effect similar to that of diazepam (1 mg/kg), characterized by an increase in the time spent in the center; also increased entries and distance into center (both acute and chronic use) | 2.5 mg/kg - p<0.05; 5 mg/kg - p=0.053 |  |
| 16 | significanlty increased mobility time and swimming duration (both acute and chronic use) | p<0.01 |  |
| 17 | significantly reduced immobility in WT mice | p<0.05 |  |
| 18 | untreated 5-HT7−/− (KO) mice showed reduced immobility compared with 5-HT7+/+ (WT) mice in the forced swim test. | p<0.001 |  |
| 18 | SB-269970 reduced immobility in 5-HT7+/+ mice but had no additional effect in 5-HT7−/− mice (already reduced). | p<0.001 |  |
| 18 | untreated 5-HT7−/− mice showed decreased immobility as compared with 5-HT7+/+ mice | p<0.001 |  |
| 18 | SB-269970 reduced immobility in 5-HT7+/+ mice but had no additional effect in 5-HT7−/− mice (already reduced). | p<0.01 |  |
| 19 | in untreated animals there was a 55% reduction in the number of marbles buried by 5-HT7−/− mice compared to 5-HT7+/+ mice | p<0.01 |  |
| 19 | 5-HT7+/+ mice treated with SB-269970 buried fewer marbles than vehicle-treated 5-HT7+/+ mice; no additional effect in 5-HT7−/− mice (already reduced). | p<0.01 |  |
| 20 | single administration, compound 22 at dose of 3 mg/kg reversed MK-801-induced memory impairment | p<0.05 |  |
| 20 | reduced the immobility time of animals by about 34% at dose of 10 mg/kg; however also reduced overall locomotion | p<0.001 |  |
| 21 | showed antidepressant effect only at a 25mg/kg (dose–response curves were U-shaped) | p<0.05 |  |
| 22 | significant reduction in the immobility time (159.1 vs 118.9 s) | NR |  |
| **No.** | **Results** | **P-Value** | **Other notes** |
| 23 | significant reduction in immobility at 0.3 and 1.0 mg/kg but not higher doses (3 and 10 mg/kg) | p<0.01 |  |
| 24 | significantly reduced immobility time at 10 and 20 mg/kg | 10mg/kg - p<0.01; 20mg/kg - p<0.0001 | overall no changes to general locomotor activity |
| 24 | signficanlty reduced immobility time at 20mg/kg | p<0.0001 | overall no changes to general locomotor activity |
| 24 | signficanlty reduced immobility time at 10mg/kg | p<0.0001 | overall no changes to general locomotor activity |
| 24 | signficanlty reduced immobility time at 20mg/kg | p<0.0001 | overall no changes to general locomotor activity |
| 24 | significantly increased number of punished crossings at 20mg/kg | p<0.05 | overall no changes to general locomotor activity |
| 24 | significantly increased number of punished crossings at 40mg/kg | p<0.01 | overall no changes to general locomotor activity |
| 24 | significantly increased number of punished crossings at 20mg/kg | p<0.05 | overall no changes to general locomotor activity |
| 24 | not significant | NS | overall no changes to general locomotor activity |
| 25 | at dose of 5mg/kg significantly reduced immobility time compared to the control group by roughly 36% | p<0.01 |  |
| 25 | significant antidepressant-like activity at the doses of 5 mg/kg and 10 mg/kg shortening immobility time by about 35% and 26%, respectively, compared to appropriate control group | p<0.001 |  |
| **No.** | **Results** | **P-Value** | **Other notes** |
| 26 | non-significant increase in time spent in the light side, and significant increase in mean time of each visit spent in light side | p<0.05 |  |
| 26 | while no differences in total distance, mice treated with DUQ0002I spent significantly more time in the open arm and greater percent of total distance in open arm | p<0.05 |  |
| 26 | no significant differences | NS |  |
| 26 | significant decrease in immobility time | p<0.01 |  |
| 27 | actute adminstration of 10mg/kg showed decreased immobility | p<0.05 |  |
| 27 | not significant | NS |  |
| 27 | AS-19 at 10mg/kg significnantly increased immobility time compared to PWD treated with vehicle | p<0.05 | increased depressive effects |
| 28 | no significant changes in total number of crosses or total time in open space at 1, 3, or 10 mg/kg | NS |  |
| 28 | no significant changes in total number of crosses or total time in open space at 10 or 30 mg/kg | NS |  |
| 28 | no significant changes in immobility time at 10mg/kg | NS |  |
| 28 | no significant changes in immobility time at 10 or 30 mg/kg | NS |  |
| 29 | significanlty decreased immobility time at 1mg/kg but not 10mg/kg | p<0.01 |  |
| 29 | significantly decreased immobility time at 1mg/kg | p<0.01 |  |
| 30 | fluoxetine showed significantly less visits to the center compared to vehicle, indicating a anxiogenic effect. SB-29970 did not show any changes compared to vehicle alone. It signicantly increased number of crossing when adminstered in conjuction with fluoxetine compared to fluoxetine alone - ameliorating fluoxetine's anxiogenic effect. | p<0.05 |  |
| 31 | FSL rats displayed significantly increased immobility and decreased swimming and climbing behavior compared with those of FRL rats. Lu AA21004 at 7.8 mg/kg significantly decreased the immobility time in the FSL rats but not in the FRL rats | p<0.05 | LuAA21004 did not significantly reduce locomotor activity in either FSL or FRL rats in open field test |
| 31 | dose-dependent anxiolytic-like effect; doses of 3.9 and 7.9 mg/kg led to significantly less fear-induced ultrasonic vocalisation compared with vehicle | p<0.05 |  |
| **No.** | **Results** | **P-Value** | **Other notes** |
| 32 | 1.25 and 2.5 mg/kg (but not 5mg/kg) produced significant decrease in immobility | 1.25mg/kg - p<0.0001; 2.5mg/kg - p<0.01 |  |
| 33 | significantly decreased immobility time at 0.1mg/kg | p < 0.0001 |  |
| 33 | significantly decreased immobility time at 0.1mg/kg | p < 0.0001 |  |
| 33 | significantly decreased immobility time at 0.03mg/kg compared to antidepressant alone | ESC p<0.0001; BUP p<0.05 |  |
| 33 | significantly decreased immobility time at 0.03mg/kg compared to antidepressant alone | ESC p<0.01; BUP p<0.001 |  |
| 34 | 2.5 and 5 mg/kg significantly decreased immobility time | p<0.01 |  |
| 34 | 1.25, 2.5, and 5 mg/kg significantly decreased immobility time | p<0.01 |  |
| 34 | 5 mg/kg significantly decreased immobility and increased swimming behaviour | p<0.01 |  |
| 34 | 1.25 and 2.5 mg/kg significantly decreased immobility time and increased the time of swimming | 1.25mg/kg - p<0.05; 2.5mg/kg - p<0.01 |  |
| 34 | no significant changes in exploratory activity | NS |  |
| 34 | no significant changes in exploratory activity | NS |  |
| 34 | 2.5 and 5 mg/kg significantly and dose-dependently increased the number of punished crossings | 2.5mg/kg - p<0.01; 5mg/kg - p<0.0001 |  |
| **No.** | **Results** | **P-Value** | **Other notes** |
| 34 | 2.5 and 5 mg/kg significantly and dose-dependently increased the number of punished crossings | 2.5mg/kg - p<0.05; 5mg/kg - p<0.001 |  |
| 35 | both doses (0.625 and 1.25 mg/kg) injected for 21 days significantly decreased immobility and increased swimming time | 0.625mg/kg - p<0.05; 1.25mg/kg - p<0.01 |  |
| 35 | not significant | NS |  |
| 35 | in retention trial HBK-15 (0.625 mg/kg) significantly increased latency time | p<0.05 |  |
| 36 | 2.5 mg/kg signifcantly reduced immobility time compared to chronic stressed mice with vehicle (saline) | p<0.01 |  |
| 36 | 2.5 mg/kg signifcantly increased sucrose consumption compared to stressed mice treated with salie | p<0.01 |  |
| 36 | 2.5 mg/kg signifcantly increased time in open arm and number of open arm entries compared to chronic stressed mice with vehicle (saline) | p<0.01 |  |
| 37 | did not significanlty reduce immobility time | NS |  |
| 37 | 1.25 mg/kg signifcantly reduced immobility time | p<0.001 |  |
| 37 | did not signficantly increase sucrose consumption | NS |  |
| 37 | 1.25mg/kg (but not 0.625) signficantly increased sucrose consumption compared to stressed mice treated with vehicle | p<0.01 |  |
| **No.** | **Results** | **P-Value** | **Other notes** |
| 37 | 2.5 mg/kg significantly increased entries to open arm | p<0.001 |  |
| 37 | 1.25 mg/kg significantly increased entries to and time in open arm | p<0.05 |  |
| 38 | increased immobility is normalised in the transgenic mouse was normalised by treatment with SB269970 (10 mg/kg) | p=0.02 |  |
| 39 | minimum effective dose >20mg/kg | NR |  |
| 39 | minimum effective dose <2.5mg/kg | NR |  |
| 39 | minimum effective dose <10mg/kg | NR |  |
| 39 | minimum effective dose >10mg/kg | NR |  |
| 39 | miminum effective dose <1mg/kg | NR |  |
| 39 | minimum effective dose >10mg/kg | NR |  |
| 40 | minimum effective dose >20mg/kg | NR |  |
| 40 | minimum effective dose <2.5mg/kg | NR |  |
| 40 | minimum effective dose >10mg/kg | NR |  |
| 40 | minimum effective dose <1mg/kg | NR |  |
| 40 | no signficant differences | NS |  |
| 40 | no signficant differences | NS |  |
| 41 | significantly reduced immobility time at 40mg/kg | p<0.05 |  |
| 41 | significantly reduced immobility time at 40mg/kg | p<0.05 |  |
| **No.** | **Results** | **P-Value** | **Other notes** |
| 41 | significantly reduced immobility time at 40mg/kg | p<0.05 |  |
| 41 | not significant | NS |  |
| 42 | significantly reduced immobility time at 2.5mg/kg | p<0.01 | did not reduce overall locomotor activity |
| 42 | not significant | NS |  |
| 42 | significantly reduced immobility time at 2.5 and 5mg/kg | 2.5mg/kg - p<0.01; 5mg/kg - p<0.001 | did not reduce overall locomotor activity |
| 43 | (0.3, 1 and 3 μg) produced an anxiolytic-like effect increasing (by 108%, 201% and 122%, respectively) the number of shocks accepted during the experimental session in the conflict drinking test | 0.3 & 3μg - p<0.05; 1μg - p<0.01 | did not reduce overall locomotor activity |
| 43 | significantly reduced immobility time at 1 and 3 μg | p<0.01 | did not reduce overall locomotor activity |
| 44 | 1 mg/kg (but not 0.5 or 2.5 mg/kg) exerted anxiolytic-like activity having significantly increased (by 211%) the number of shocks accepted | p<0.01 | did not reduce overall locomotor activity |
| 44 | 0.5mg/kg significantly increased time spent in open arm and number of entries. 1mg/kg increased number of entries.not significant at 0.25 or 2.5 mg/kg | p<0.05 |  |
| 44 | 1 mg/kg (but not 0.5 or 2.5 mg/kg) significantly increased (by 70%) the number of punished crossings | p<0.05 |  |
| 44 | 10 mg/kg (but not 5 or 20 mg/kg) significantly reduced (by 29%) the immobility time | p<0.05 |  |
| 44 | 5 mg/kg (but not 2.5, 10 or 20 mg/kg) produced a statistically significant reduction (by 28%) in the immobility time | p<0.01 |  |
| 45 | low doses of citalopram, imipramine, desipramine, and moclobemide did not reduce immobility time in mice on their own, but did when coadministered with SB-269970. | p<0.05 |  |
| 46 | shortened immobility time in the FST and decreased activity in the spontaneous locomotor activity test at 10 mg/kg dose | p<0.01 |  |
| **No.** | **Results** | **P-Value** | **Other notes** |
| 46 | not significant | NS |  |
| 46 | not significant | NS |  |
| 47 | significantly decreased immobility time at 10mg/kg (but not 2.5, 5 or 20) | not listed |  |
| 47 | not significant | NS |  |
| 47 | not significant | NS |  |
| 47 | significantly increased shocked crossings at 5mg/kg (but not 2.5 or10) | not listed |  |
| 48 | significantly reduced immobility time at 2.5 and 5mg/kg (but not 1.25 or 10) | p<0.05 |  |
| 48 | significantly increased shocked crossings at 2.5mg/kg (but not 1 or 5) | p<0.01 |  |
| 49 | significantly reduced immobility time at 10mg/kg (but not 5 or 20) | p<0.05 |  |
| 50 | signficanlty reduced immobility time at 30 mg/kg (but not 10 or 20) | p<0.001 |  |
| 50 | signficantly increased time spent in and entries into open arm at 2.5 and 10 mg/kg (but not 5). | p<0.05-p<0.001 |  |
| 51 | signficanlty reduced immobility time at 5 and 10 mg/kg (but not 2.5) | p<0.001 |  |
| 51 | signficanlty reduced immobility time at 5, 10 and 20 mg/kg (but not 2.5) | 5mg/kg - p<0.01; 10&20mg/kg - p<0.001 |  |
| 52 | Compound 7 (5 mg/kg) significantly reduced the immobility time of mice | p<0.01 |  |
| **No.** | **Results** | **P-Value** | **Other notes** |
| 52 | potency of the antidepressant-like activity of compound 10 was similar to that of the selective 5-HT7 receptor antagonist SB-269970, which exerted its characteristic antidepressant effect at one medium dose (10 mg/kg) | p<0.01 |  |
| 52 | not significant | NS |  |
| 52 | compound 10 displayed anxiolytic-like properties (1.25 mg/kg) increasing the number of punished crossings by 75% | p<0.05 |  |
| 52 | acute administration of 7 dose-dependently ameliorated PCP-induced memory deficits in rats. All the tested compounds reversed episodic memory decline at a dose of 1 mg/kg | p<0.001 |  |
| 52 | acute administration of 10 dose-dependently ameliorated PCP-induced memory deficits in rats. All the tested compounds reversed episodic memory decline at a dose of 1 mg/kg | p<0.001 |  |
