# Supplementary material for: The 5-HT7 receptor system as a treatment target for mood and anxiety disorders: A systematic review
Source: J Psychopharmacol. 2023 Nov 23;37(12):1167–81. doi: 10.1177/02698811231211228 (PMC10714716; doi:10.1177/02698811231211228)
Supplement: sj-docx-2-jop-10.1177_02698811231211228 – Supplemental material for The 5-HT7 receptor system as a treatment target for mood and anxiety disorders: A systematic review [file sj-docx-2-jop-10.1177_02698811231211228.docx]

| **No.** | **Author / Year** | **Compound Name** | **Chemical Name** | **Listed in paper** | **Listed PDSP database** | **Source** |
| --- | --- | --- | --- | --- | --- | --- |
| 1 | Abbas 2009 | Amisulpride | 4-amino-N-[(1-ethylpyrrolidin-2-yl)methyl]-5-ethylsulfonyl-2-methoxybenzamide | yes | yes | paper (database cites paper; also includes certified data) |
| 2 | Adriani 2012 | LP-211 | N-(4-cyanophenylmethyl)-4-(2-diphenyl)-1-piperazinehexanamide | no | yes | Hedlund 2010 (database) |
| 2 | Adriani 2012 | LP-378 | N-(4-Trifluorophenylmethyl)-4-(2-diphenyl)-1-piperazinehexanamide | yes |  | paper |
| 3 | Balcer 2019 | N/A - knock out mice |  |  |  |  |
| 4 | Bonaventure 2007 | SB-269970 | (2R)-1-[(3-hydroxyphenyl)sulfonyl]-2-[2-(4-methyl-1-piperidinyl)ethyl]-pyrrolidine | no | yes | Lovell 2000 (database) |
| 5 | Bonaventure 2012 | JNJ-18038683 | (3-(4-chlorophenyl)-1,4,5,6,7,8- hexahydro-1-(phenylmethyl)pyrazolo[3,4-d]azepine 2-hydroxy-1,2,3-propanetricarboxylate) | some | no | paper |
| 6 | Canale 2015 | Compound 32 | (S)-2-(5-(4-([1,1'-Biphenyl]-2-yl)piperazin-1-yl)pentanoyl)-1,2,3,4-tetrahydroisoquinoline-3-carboxamide | some | no | paper |
| 7 | Canale 2016a | Compound 17 | 1-Methyl-N-(1-{[2-(t-butyl-2-yl)phenoxy]ethyl}piperidin-4-yl)-N-methyl-1H-pyrazole-sulfonamide | some | no | paper |
| 7 | Canale 2016a | Compound 20 | 1-Methyl-N-{1-[2-(biphenyl-2-yloxy)ethyl]piperidin-4-yl}-N-methyl-1H-pyrazole-4-sulfonamide | some | no | paper |
| 7 | Canale 2016a | Compound 31 | (1-methyl-N-{1-[2-(2-(t-butyl)phenoxy)ethyl]piperidin-4-yl}-N-cyclopropylmethyl-1H-pyrazole-4-sulfonamide) | some | no | paper |
| 7 | Canale 2016a | Compound 33 | 1-Methyl-N-{1-[2-(biphenyl-2-yloxy)ethyl]piperidin-4-yl}-N-cyclopropylmethyl-1H-pyrazole-4-sulfonamide | some | no | paper |
| 8 | Canale 2016b | PZ-1417 (27) | 3-fluoro-N-{1-[2-(2-cyclopentylphenoxy)ethyl]piperidin-4-yl}-benzenesulfonamide; PZ-1417 | some | no | paper |
| 8 | Canale 2016b | PZ-1150 (35) | 4-fluoro-N-(1-{2-[(propan-2-yl)phenoxy]ethyl}-8-azabicyclo[3.2.1]octan-3-yl)-benzenesulfonamide | some | no | paper |
| 9 | Canale 2017 | Compound 20 | 4-Fluoro-N-(1-{3-[(2-isopropylphenoxy)]propyl}piperidin-4-yl)benzenesulfonamide | some | no | paper |
| 9 | Canale 2017 | Compound 25 | 3-chloro-N-{1-[3-(1,1-biphenyl-2- yloxy)2-hydroxypropyl]piperidin-4-yl}benzenesulfonamide | some | no | paper |
| **No.** | **Author / Year** | **Compound Name** | **Chemical Name** | **Listed in paper** | **Listed PDSP database** | **Source** |
| 10 | Cates 2013 | Lurasidone | (3aR,4S,7R,7aS)-2-{(1R,2R)-2-[4-(1,2-benzisothiazol-3-yl)piperazin-1-ylmethyl] cyclohexylmethyl}hexahydro-4,7-methano-2H-isoindole-1,3-dione | no | yes | Ishibashi 2010 (database) |
| 11 | Chlon-Rzepa 2013 | Compound 21 | 7-Benzyl-8-((4-(4-(3-chlorophenyl)piperazin-1- yl)butyl)amino)-1,3-dimethyl-1H-purine2,6(3H,7H)-dione hydrochloride | some | no | paper |
| 11 | Chlon-Rzepa 2013 | Compound 42 | 8-(3-(4-(4-Fluorophenyl)piperazin-1-yl)propoxy)-1,3-dimethyl-7-(3-phenylpropyl)- 1H-purine-2,6(3H,7H)-dione hydrochloride | some | no | paper |
| 12 | Delcourte 2017 | Asenapine | (3aRS,12bRS)-rel-5-Chloro-2,3,3a,12b-tetrahydro- 2-methyl-1H-dibenz[2,3:6,7]oxepino[4,5-c]pyrrole | no | yes | PDSP certified (database) |
| 13 | Gu 2017 | Compound 8j | 3-(4-(3,4-dichlorophenethyl)piperazin-1-yl)benzo[d]isothiazole hydrochloride | some | no | paper |
| 14 | Gu 2018 | Compound 21n | 5-Fluoro-3-(3-(4-(5-fluoro-[1,1′-biphenyl]-2-yl)piperazin-1-yl)propyl)-1H-indole hydrochloride | some | no | paper |
| 15 | Gu 2019 | Compound 19a | 5-fluoro-3-(3-(4-(5-fluoro-[1,1'-biphenyl]-2-yl)piperidin-1-yl)propyl)-1H-indole | some | no | paper |
| 16 | Guilloux 2013 | Vortioxitine | 1-[2-(2,4-dimethyl-phenylsulfanyl)-phenyl]-piperazine | some | no | Bang-Andersen 2011, Mork 2012, Westrich 2012 (cited in paper) |
| 17 | Guscott 2005 | SB-258719 (and KO mice) | (1R)-3,N-dimethyl-N-[1-methyl-3-(4-methylpiperidin-1-yl)propyl]benzenesulfonamide | no | yes | Frobes 1998 (database) |
| 18 | Hedlund 2005 | SB-269970 (and 5HT7 KO) | (2R)-1-[(3-hydroxyphenyl)sulfonyl]-2-[2-(4-methyl-1-piperidinyl)ethyl]-pyrrolidine | no | yes | Lovell 2000 (database) |
| 19 | Hedlund 2007 | SB-269970 (and 5HT7 KO) | (2R)-1-[(3-hydroxyphenyl)sulfonyl]-2-[2-(4-methyl-1-piperidinyl)ethyl]-pyrrolidine | no | yes | Lovell 2000 (database) |
| 20 | Jankowska 2020 | Compound 22 | N-(4-isopropylphenyl)-5-(4-(2-methoxyphenyl)piperazin-1-yl)pentanamide hydrochloride | some | no | paper |
| 21 | Kim 2014 | Compound 1-8 | N-((2′-Chlorobiphenyl-2-yl)methyl)-5-(4-(2-methoxyphenyl)piperazin-1-yl)pentanamide | yes | no | paper |
| 22 | Kim 2016 | Compound 28 | 1-(9H-carbazol-9-yl)-6-(4-(2-methoxyphenyl)piperazin-1-yl)hexan-1-one hydrochloride | some | no | paper |
| 23 | Kolaczkowski 2014 | ADN-1184 | 1-(1-adamantyl)-N-1-naphthyl-4-nitro-1H-pyrazole-3-carboxamide | yes | no | paper |
| **No.** | **Author / Year** | **Compound Name** | **Chemical Name** | **Listed in paper** | **Listed PDSP database** | **Source** |
| 24 | Kucwaj-Brysz 2018 | Compound 5 | 5-Phenyl-3-(3-(4-(2-methoxyphenyl)piperazin-1-yl)-2-hydroxypropyl)-5-methylimidazolidine-2,4-dione hydrochloride | some | no | paper |
| 24 | Kucwaj-Brysz 2018 | Compound 6 | 5-Phenyl-3-(3-(4-(2-cyanophenyl)piperazin-1-yl)-2-hydroxypropyl)-5-methylimidazolidine-2,4-dione hydrochloride | some | no | paper |
| 24 | Kucwaj-Brysz 2018 | Compound 7 | 5-(4-Bromophenyl)-3-(3-(4-(2-methoxyphenyl)piperazin-1-yl)-2-hydroxypropyl)-5-methylimidazolidine-2,4-dione hydrochloride | some | no | paper |
| 24 | Kucwaj-Brysz 2018 | Compound 8 | 5-(4-Bromophenyl)-3-(3-(4-(2-cyanophenyl)piperazin-1-yl)-2-hydroxypropyl)-5-methylimidazolidine-2,4-dione hydrochloride | some | no | paper |
| 25 | Latacz 2018 | MF-8 | (5-(4-fluorophenyl)-3-(2-hydroxy-3-(4-(2-methoxyphenyl)piperazin-1-yl)propyl)-5-methylhydantoin) | some | no | paper |
| 26 | Lax 2018 | DUQ0002-I |  | some | no | paper |
| 27 | Li 2013 | Vortioxitine | 1-[2-(2,4-dimethyl-phenylsulfanyl)-phenyl]-piperazine | no | no | paper (cites Bang-Andersen 2011, Mork 2012, Westrich 2012) |
| 27 | Li 2013 | SB-269970 | (2R)-1-[(3-hydroxyphenyl)sulfonyl]-2-[2-(4-methyl-1-piperidinyl)ethyl]-pyrrolidine | no | yes | Lovell 2000 (database) |
| 27 | Li 2013 | AS-19 | (2S)-N,N-dimethyl-5-(1,3,5-trimethylpyrazol-4-yl)-1,2,3,4-tetrahydronaphthalen-2-amine | no | yes | Perry 2020 (database) |
| 28 | Maxwell 2019 | DR-4004 |  | no | no | Meneses 2004 (cited in paper) |
| 28 | Maxwell 2019 | SB-269970 | (2R)-1-[(3-hydroxyphenyl)sulfonyl]-2-[2-(4-methyl-1-piperidinyl)ethyl]-pyrrolidine | no | yes | Lovell 2000 (database) |
| 29 | Medina 2014 | Compound 6 | 1-[(3E)-6-(3,4-Dihydroisoquinolin-2(1H)-yl)hex-3-en-1-yl]-1,3-dihydro-2H-indol-2-one | some | no | paper |
| 30 | Mnie-Filali 2011 | SB-269970 | (2R)-1-[(3-hydroxyphenyl)sulfonyl]-2-[2-(4-methyl-1-piperidinyl)ethyl]-pyrrolidine | no | yes | Lovell 2000 (database) |
| 31 | Mork 2012 | Lu AA21004 | 1-[2-(2,4-Dimethylphenylsulfanyl)phenyl]piperazine | some | no | Bang-Anderson 2011 (cited in paper) |
| 32 | Partyka 2017 | Compound 16 | 4-({4-(2-[4-(3-Chlorophenyl)piperazin-1-yl]ethyl)piperidin-1-yl}sulfonyl)isoquinoline | some | yes | paper (database cites paper) |
| **No.** | **Author / Year** | **Compound Name** | **Chemical Name** | **Listed in paper** | **Listed PDSP database** | **Source** |
| 32 | Partyka 2017 | Compound 21 | 5-({4-(2-[4-(2,3-dichlorophenyl)piperazin-1-yl]ethyl)piperidin-1-yl}sulfonyl)quinoline | some | yes | paper (database cites paper) |
| 33 | Partyka 2019 | PZ-1433 (compound 20 in Canale 2017) | (4-Fluoro-N-(1-{3-[(2-isopropylphenoxy)]propyl}piperidin-4 yl)benzenesulfonamide) | no | no | Canale 2017 (cited in paper) |
| 33 | Partyka 2019 | ADN-1184 | 1-(1-adamantyl)-N-1-naphthyl-4-nitro-1H-pyrazole-3-carboxamide | no | no | Kolaczkowski 2014a (cited in paper) |
| 34 | Pytka 2015 | HBK-14 | 1-{2-[2-(2,6-dimethlphenoxy)ethoxy]ethyl}-4-(2-methoxyphenyl)piperazynine hydrochloride | some | no | paper |
| 34 | Pytka 2015 | HBK-15 | 2-[2-(2-chloro-6-methylphenoxy)ethoxy]ethyl-4-(2- methoxyphenyl)piperazynine dihydrochloride | some | no | paper |
| 35 | Pytka 2017a | HBK-15 | 2-[2-(2-chloro-6-methylphenoxy)ethoxy]ethyl-4-(2- methoxyphenyl)piperazynine dihydrochloride | some | no | paper |
| 36 | Pytka 2017b | HBK-14 | 1-{2-[2-(2,6-dimethlphenoxy)ethoxy]ethyl}-4-(2-methoxyphenyl)piperazynine hydrochloride | some | no | Pytka 2015 (cited in paper) |
| 36 | Pytka 2017b | HBK-15 | 2-[2-(2-chloro-6-methylphenoxy)ethoxy]ethyl-4-(2- methoxyphenyl)piperazynine dihydrochloride | some | no | Pytka 2015 (cited in paper) |
| 37 | Pytka 2018 | HBK-14 | 1-[(2,6-Dimethylphenoxy)ethoxyethyl]-4-(2-methoxyphenyl)piperazine hydrochloride | no | no | Pytka 2015 (cited in paper) |
| 37 | Pytka 2018 | HBK-15 | 1-[(2-chloro-6-methylphenoxy)ethoxyethyl]-4-(2-methoxyphenyl)piperazine hydrochloride | no | no | Pytka 2015 (cited in paper) |
| 38 | Stroth 2015 | SB-269970 | (2R)-1-[(3-hydroxyphenyl)sulfonyl]-2-[2-(4-methyl-1-piperidinyl)ethyl]-pyrrolidine | no | yes | Lovell 2000 (database) |
| 39 | Volk 2008 | Compound 9e' | 3-{4-[4-(4-Chlorophenyl)-piperazin-1-yl]-butyl}-3-ethyl-6-fluoro-1,3-dihydro-2H-indol-2-one | yes | no | paper |
| 39 | Volk 2008 | Compound 12d | 3-{4-[4-(4-Chloro-phenyl)-piperazin-1-yl]-butyl}-1,3-dihydro-2H-indol-2-one | yes | no | paper |
| 39 | Volk 2008 | Compound12e | 5-Fluoro-3-[4-(4-phenyl-piperazin-1-il)-butyl]-1,3-dihydro-2H-indol-2-one | yes | no | paper |
| 40 | Volk 2011 | Compound 1a | 3-{4-[4-(4-chlorophenyl)piperazin-1-yl]butyl}-3-ethyl-6-fluoro-1,3-dihydro-2H-indol-2-one | some | no | paper |
| 40 | Volk 2011 | Compound 2a |  | some | no | paper |
| 41 | Wang 2019 | Compound 7a | 5-fluoro-3-(1-(2-((5-fluoro-[1,1'-biphenyl]-2-yl)oxy)ethyl)piperidin-4-yl)-1H-indole | some | no | paper |
| **No.** | **Author / Year** | **Compound Name** | **Chemical Name** | **Listed in paper** | **Listed PDSP database** | **Source** |
| 41 | Wang 2019 | Compound 15g | 3-(1-(2-((3',5-difluoro-[1,1'-biphenyl]-2-yl)oxy)ethyl)piperidin-4-yl)-5-fluoro-1H-indole | some | no | paper |
| 42 | Waszkielewicz 2015 | Compound 2 | 1-[(2,5-dimethylphenoxy)propyl]-4-(2-methoxyphenyl)piperazine hydrochloride | some | no | paper |
| 42 | Waszkielewicz 2015 | Compound 3 | 1-[(2,3,5-trimethylphenoxy)propyl]-4-(2-methoxyphenyl)piperazine hydrochloride | some | no | paper |
| 42 | Waszkielewicz 2015 | Compound 6 | 1-[(2-chloro-6-methylphenoxy)ethoxyethyl]-4-(2-methoxyphenyl)piperazine hydrochloride | some | no | paper |
| 43 | Wesolowska 2006a | SB-269970 | (2R)-1-[(3-hydroxyphenyl)sulfonyl]-2-[2-(4-methyl-1-piperidinyl)ethyl]-pyrrolidine | no | yes | Lovell 2000 (database) |
| 44 | Wesolowska 2006b | SB-269970 | (2R)-1-[(3-hydroxyphenyl)sulfonyl]-2-[2-(4-methyl-1-piperidinyl)ethyl]-pyrrolidine | no | yes | Lovell 2000 (database) |
| 45 | Wesolowska 2007 | SB-269970 (plus other antidepressants) | (2R)-1-[(3-hydroxyphenyl)sulfonyl]-2-[2-(4-methyl-1-piperidinyl)ethyl]-pyrrolidine | no | yes | Lovell 2000 (database) |
| 46 | Wrobel 2019 | MW005 | 1-{4-[4-(5-fluoro-1H-indol-3-yl)piperidin-1-yl]butyl}-3-(1H-indol-3-yl)pyrrolidine-2,5-dione | some | yes | paper (database cites paper) |
| 46 | Wrobel 2019 | compound 4A | 1-{4-[4-(1H-indol-3-yl)piperidin-1-yl]butyl}-3-(5-methoxy-1H-indol-3-yl)pyrrolidine-2,5-dione | some | yes | paper (database cites paper) |
| 46 | Wrobel 2019 | compound 4J | 1-{4-[4-(1H-indol-3-yl)piperidin-1-yl]butyl}-3-(5-fluoro-1H-indol-3-yl)pyrrolidine-2,5-dione | some | yes | paper (database cites paper) |
| 47 | Zagorska 2015 | Compound 8 | 7-Phenyl-8-[4-(N4-2′-hydroxyphenyl)-piperazin-N1-yl-butyl]-1,3-dimethyl-(1H,8H)-imidazo[2,1-f]purine-2,4-dione | some | no | paper |
| 47 | Zagorska 2015 | Compound 9 | 7-Phenyl-8-[5-(N4-2′-hydroxyphenyl)-piperazin-N1-yl-pentyl]-1,3-dimethyl-(1H,8H)-imidazo[2,1-f]purine-2,4-dione | some | no | paper |
| 48 | Zagorska 2016 | Compound 9 | 8-(5-(4-(2-fluorophenyl)piperazin-1-yl)pentyl)-1,3,7-trimethyl-1H-imidazo[2,1-f]purine-2,4(3H,8H)-dione | some | no | paper |
| 49 | Zajdel 2011 | Compound 54 | (N-Ethyl-N-[4-(1,2,3,4,4a,5,6,7,8,8a-decahydroisoquinolin-2-yl)butyl]-8-quinolinesulfonamide) | some | no | paper |
| 50 | Zajdel 2012 | Compound 36 | (4-(4-{2-[4-(4-chloro-phenyl)-piperazin-1-yl]-ethyl}-piperidine-1-sulfonyl)-isoquinoline) | some | no | paper |
| 51 | Zajdel 2013 | Compound 33 | (N-(3-(4-(2,3-dichlorophenyl)piperazin-1-yl)propyl)quinoline-7-sulfonamide) | some | no | paper |
| 51 | Zajdel 2013 | Compound 39 | (N-(4-(4-(2,3-dichlorophenyl)piperazin-1-yl)butyl)isoquinoline-3-sulfonamide) | some | no | paper |
| **No.** | **Author / Year** | **Compound Name** | **Chemical Name** | **Listed in paper** | **Listed PDSP database** | **Source** |
| 52 | Zajdel 2015 | Compound 7 (PZ-766) | 4-fluoro-N-(1-{2-[(propan-2-yl)phenoxy]ethyl}piperidin-4-yl) benzenesulfonamide | some | no | paper |
| 52 | Zajdel 2015 | Compound 10 (PZ-1404) | (3-fluoro-N-(1-{2-[(propan-2-yl)phenoxy]ethyl}piperidin-4-yl)-benzenesulfonamide) | some | no | paper |

| **No.** | **Notes** | **SERT** | **NET** | **5-HT1A** | **5-HT1B** | **5-HT1D** | **5-HT1E** | **5-HT1F** | **5-HT2A** | **5-HT2B** | **5-HT2C** | **5-HT3** | **5-HT4** | **5-HT5a** | **5-HT6** |
| --- | --- | --- | --- | --- | --- | --- | --- | --- | --- | --- | --- | --- | --- | --- | --- |
| 1 |  | >10,000 | >10,000 | >10,000 | 1744 | 1341 | >10,000 |  | 8304 | 13 | >10,000 | >10,000 |  | >10,000 | 4154 |
| 2 |  | 812 |  | 379 | 215 | 394 | > 10,000 |  | 626 | 67 | 91 | > 10,000 |  | 178 | 1,571 |
| 2 |  | >10,000 |  | 77 | 47 | 75 | >10,000 |  | 504 | 56 | 789 | >10,000 | 3092 | 929 | 2,046 |
| 3 |  |  |  |  |  |  |  |  |  |  |  |  |  |  |  |
| 4 |  |  |  | >10,000 | 1,000 | 1,584.89 | 6,309.57 | 3,162.28 | >10,000 | >10,000 | >10,000 |  | 1,258.93 | 63.095734 | 6,309.57 |
| 5 | NOT KI values - pKi values | <5 | <5 | 6.5 | 6.9 | 6.2 |  |  | 7.03 | 6.81 | 7.06 | <5 | <5 | 5.5 | 7.2 |
| 6 |  |  |  | 66 |  |  |  |  | 594 |  |  |  |  |  | 166 |
| 7 |  | (<20% at @ 1 μM) |  | 9626 |  |  |  |  | 557 |  |  |  |  |  |  |
| 7 |  | (<20% at @ 1 μM) |  | 80 |  |  |  |  | 58 |  |  |  |  |  |  |
| 7 |  | (<20% at @ 1 μM) |  | 17,770 |  |  |  |  | 1479 |  |  |  |  |  |  |
| 7 |  | (<20% at @ 1 μM) |  | 159 |  |  |  |  | 30 |  |  |  |  |  |  |
| 8 |  |  |  | 545 |  |  |  |  | 303 |  |  |  |  |  | 281 |
| 8 |  |  |  |  |  |  |  |  | 1295 |  |  |  |  |  | 559 |
| 9 |  |  |  | 2081 |  |  |  |  | 1352 |  |  |  |  |  | 268 |
| 9 |  | low (<40% @ 1 μM) |  | 1329 |  |  |  |  | 2115 |  |  |  |  |  | 295 |
| **No.** | **Notes** | **SERT** | **NET** | **5-HT1A** | **5-HT1B** | **5-HT1D** | **5-HT1E** | **5-HT1F** | **5-HT2A** | **5-HT2B** | **5-HT2C** | **5-HT3** | **5-HT4** | **5-HT5a** | **5-HT6** |
| 10 |  |  |  | 6.75 |  |  |  |  | 2.03 |  |  |  |  |  |  |
| 11 |  |  |  | 21 ± 3 |  |  |  |  | 68 ± 13 |  |  |  |  |  | > 1 µM |
| 11 |  |  |  | 34 ± 2 |  |  |  |  | 210 ± 31 |  |  |  |  |  | 717 ± 15 |
| 12 |  |  |  | 4.7 | 55 | 6.3 | 71 |  | 0.16 | 0.21 | 0.24 | 336 |  | 403/540 | 0.62 |
| 13 |  | RUI, IC50 = 100 nM |  | 0.84 |  |  |  |  |  |  |  |  |  |  |  |
| 14 |  | RUI, IC50 = 25 nM |  | 28 |  |  |  |  |  |  |  |  |  |  |  |
| 15 |  | RUI, IC50 = 14 nM |  | 12 |  |  |  |  |  |  |  |  |  |  |  |
| 16 |  | 1.6 |  | 15 | 33 | 54 |  |  |  |  |  | 3.7 |  |  |  |
| 17 |  |  |  | 7943.28 | 5011.87 | 3162.28 | >10,000 | 6309.57 | >10,000 | 5011.87 | >10,000 |  | >10,000 |  | >10,000 |
| 18 |  |  |  | >10,000 | 1,000 | 1,584.89 | 6,309.57 | 3,162.28 | >10,000 | >10,000 | >10,000 |  | 1,258.93 | 63.095734 | 6,309.57 |
| 19 |  |  |  | >10,000 | 1,000 | 1,584.89 | 6,309.57 | 3,162.28 | >10,000 | >10,000 | >10,000 |  | 1,258.93 | 63.095734 | 6,309.57 |
| 20 |  |  |  | 8 |  |  |  |  |  |  |  |  |  |  |  |
| 21 |  |  |  | 20 | 131 | 418 |  |  | 478 |  | 26 | >10,000 |  | 1178 | 1517 |
| 22 |  |  |  | 549 | 845 | 1280 |  |  | 1265 | 419 | 588 | 18% @ 10 μM |  | 34% @ 10 μM | 31% @ 10 μM |
| 23 |  |  |  | 173 |  |  |  |  | 2 |  | 630 |  |  |  | 16 |
| **No.** | **Notes** | **SERT** | **NET** | **5-HT1A** | **5-HT1B** | **5-HT1D** | **5-HT1E** | **5-HT1F** | **5-HT2A** | **5-HT2B** | **5-HT2C** | **5-HT3** | **5-HT4** | **5-HT5a** | **5-HT6** |
| 24 |  |  |  | 421 |  |  |  |  |  |  |  |  |  |  |  |
| 24 |  |  |  | 585 |  |  |  |  |  |  |  |  |  |  |  |
| 24 |  |  |  | 85 |  |  |  |  |  |  |  |  |  |  |  |
| 24 |  |  |  | 140 |  |  |  |  |  |  |  |  |  |  |  |
| 25 |  |  |  | 121 |  |  |  |  |  |  |  | 16.5 |  |  | 10,790 |
| 26 |  | failed primary binding | >10,000 | 1746 |  |  |  |  |  |  |  |  |  |  |  |
| 27 |  | 1.6 |  | 15 | 33 | 54 |  |  |  |  |  | 3.7 |  |  |  |
| 27 |  |  |  | >10,000 | 1000 | 1584.893192 | 6309.573445 | 3162.27766 | >10,000 | >10,000 | >10,000 |  | 1258.9254 | 63.095734 | 6309.573445 |
| 27 |  |  |  | 240 |  |  |  |  |  |  |  |  |  |  |  |
| 28 | NOT KI values - pKi values |  |  | 6.7 | <6 | <6 |  |  | 7 |  | <6 | <6 |  |  |  |
| 28 |  |  |  | >10,000 | 1000 | 1584.893192 | 6309.573445 | 3162.27766 | >10,000 | >10,000 | >10,000 |  | 1258.9254 | 63.095734 | 6309.573445 |
| 29 |  |  |  | >1000 |  |  |  |  |  |  |  |  |  |  |  |
| 30 |  |  |  | >10,000 | 1000 | 1584.893192 | 6309.573445 | 3162.27766 | >10,000 | >10,000 | >10,000 |  | 1258.9254 | 63.095734 | 6309.573445 |
| 31 |  | 1.6 |  | 15 | 33 |  |  |  |  |  |  | 3.7 |  |  |  |
| 32 |  |  |  | 32 |  |  |  |  | 157 |  |  |  |  |  | 1185 |
| **No.** | **Notes** | **SERT** | **NET** | **5-HT1A** | **5-HT1B** | **5-HT1D** | **5-HT1E** | **5-HT1F** | **5-HT2A** | **5-HT2B** | **5-HT2C** | **5-HT3** | **5-HT4** | **5-HT5a** | **5-HT6** |
| 32 |  |  |  | 45 |  |  |  |  | 286 |  |  |  |  |  | 401 |
| 33 |  |  |  | 2081 |  |  |  |  | 1352 |  |  |  |  |  | 268 |
| 33 |  |  |  | 173 |  |  |  |  | 2 |  | 630 |  |  |  | 16 |
| 34 |  | 3500 |  | 41 |  |  |  |  | 264 |  |  |  |  |  |  |
| 34 |  | 529 |  | <1 nM |  |  |  |  | 109 |  |  |  |  |  |  |
| 35 |  |  |  | <1 nM |  |  |  |  | 109 |  |  |  |  |  |  |
| 36 |  | 3500 |  | 41 |  |  |  |  | 264 |  |  |  |  |  |  |
| 36 |  | 529 |  | <1 nM |  |  |  |  | 109 |  |  |  |  |  |  |
| 37 |  | 3500 |  | 41 |  |  |  |  | 264 |  |  |  |  |  |  |
| 37 |  | 529 |  | <1 nM |  |  |  |  | 109 |  |  |  |  |  |  |
| 38 |  |  |  | >10,000 | 1000 | 1584.893192 | 6309.573445 | 3162.27766 | >10,000 | >10,000 | >10,000 |  | 1258.9254 | 63.095734 | 6309.573445 |
| 39 |  |  |  | 1610 |  |  |  |  | 19.4 |  | 358 |  |  |  | 186.5 |
| 39 |  |  |  | 1800 |  |  |  |  | 17.5 |  | 690 |  |  |  | 580 |
| 39 |  |  |  | 150 |  |  |  |  | 55 |  | 1500 |  |  |  | 1800 |
| 40 |  |  |  | 1610 |  |  |  |  | 19.4 |  |  |  |  |  |  |
| 40 |  |  |  | 1800 |  |  |  |  | 17.5 |  |  |  |  |  |  |
| 41 |  | RUI, IC50 = 177 nM |  | 12 |  |  |  |  |  |  |  |  |  |  |  |
| **No.** | **Notes** | **SERT** | **NET** | **5-HT1A** | **5-HT1B** | **5-HT1D** | **5-HT1E** | **5-HT1F** | **5-HT2A** | **5-HT2B** | **5-HT2C** | **5-HT3** | **5-HT4** | **5-HT5a** | **5-HT6** |
| 41 |  | RUI, IC50 = 85 nM |  | 17 |  |  |  |  |  |  |  |  |  |  |  |
| 42 |  |  |  | <1 |  |  |  |  |  |  |  |  |  |  | 1809 |
| 42 |  |  |  | 6 |  |  |  |  |  |  |  |  |  |  | 4465 |
| 42 |  |  |  | <1 |  |  |  |  |  |  |  |  |  |  | 9617 |
| 43 |  |  |  | >10,000 | 1000 | 1584.893192 | 6309.573445 | 3162.27766 | >10,000 | >10,000 | >10,000 |  | 1258.9254 | 63.095734 | 6309.573445 |
| 44 |  |  |  | >10,000 | 1000 | 1584.893192 | 6309.573445 | 3162.27766 | >10,000 | >10,000 | >10,000 |  | 1258.9254 | 63.095734 | 6309.573445 |
| 45 |  |  |  | >10,000 | 1000 | 1584.893192 | 6309.573445 | 3162.27766 | >10,000 | >10,000 | >10,000 |  | 1258.9254 | 63.095734 | 6309.573445 |
| 46 |  |  |  | 7.5 |  |  |  |  | 71 |  |  |  |  |  | 63 |
| 46 |  | 705 |  | 6.3 |  |  |  |  | 31 |  |  |  |  |  | 81 |
| 46 |  | 51 |  | 13 |  |  |  |  | 50 |  |  |  |  |  | 29 |
| 47 |  |  |  | 6 |  |  |  |  |  |  |  |  |  |  |  |
| 47 |  |  |  | 9 |  |  |  |  |  |  |  |  |  |  |  |
| 48 |  |  |  | 2.5 |  |  |  |  |  |  |  |  |  |  |  |
| 49 |  |  |  | 1099 |  |  |  |  | 6281 |  |  |  |  |  | 1950 |
| 50 |  |  |  | 3210 |  |  |  |  | 59 |  |  |  |  |  | 16650 |
| 51 |  | 224 |  | 14 |  |  |  |  | 47 |  |  |  |  |  | 257 |
| 51 |  | 335 |  | 34 |  |  |  |  | 35 |  |  |  |  |  | 454 |
| **No.** | **Notes** | **SERT** | **NET** | **5-HT1A** | **5-HT1B** | **5-HT1D** | **5-HT1E** | **5-HT1F** | **5-HT2A** | **5-HT2B** | **5-HT2C** | **5-HT3** | **5-HT4** | **5-HT5a** | **5-HT6** |
| 52 |  |  |  | 436 |  |  |  |  |  |  |  |  |  |  | 240 |
| 52 |  |  |  | 356 |  |  |  |  |  |  |  |  |  |  | 471 |

| **No.** | **5-HT7** | **5-HT7a** | **adrenergic ⍺ 1** | **adrenergic ⍺ 1B** | **adrenergic ⍺ 2A** | **adrenergic ⍺ 2C** | **DOPAMINE D1** | **DOPAMINE D2** | **DOPAMINE D3** | **DOPAMINE D4** |
| --- | --- | --- | --- | --- | --- | --- | --- | --- | --- | --- |
| 1 | 11.5 | 135.5 | >10,000 | >10,000 | 1114 | 1540 | >10,000 | 3 | 3.5 | 2369 |
| 2 | 15 |  |  |  |  |  |  |  |  |  |
| 2 | 5.3 |  |  |  |  |  |  |  |  |  |
| 3 |  |  |  |  |  |  |  |  |  |  |
| 4 | 1.258925 |  |  | >10,000 |  |  |  | 316.227766 | 2,511.89 |  |
| 5 | 8.21 |  | 7.04 | 7 | <5 |  | 6.15 | <5 |  |  |
| 6 | 12 |  |  |  |  |  |  |  |  |  |
| 7 | 58 |  |  |  |  |  |  | 280 |  |  |
| 7 | 7 |  |  |  |  |  |  | 35 |  |  |
| 7 | 49 |  |  |  |  |  |  | 230 |  |  |
| 7 | 10 |  |  |  |  |  |  | 16 |  |  |
| 8 | 17 |  | 1525 |  |  |  |  | 322 |  |  |
| 8 | 1 |  | 339 |  |  |  |  | 60 |  |  |
| 9 | 32 |  |  |  |  |  |  | 328 |  |  |
| 9 | 33 |  | no affinity (<10% @ 1 μM) |  |  |  |  | 343 |  |  |
| **No.** | **5-HT7** | **5-HT7a** | **adrenergic ⍺ 1** | **adrenergic ⍺ 1B** | **adrenergic ⍺ 2A** | **adrenergic ⍺ 2C** | **DOPAMINE D1** | **DOPAMINE D2** | **DOPAMINE D3** | **DOPAMINE D4** |
| 10 | 0.495 |  | 47.9 |  | 40.7 | 10.8 |  | 1.68 |  |  |
| 11 | 65 ± 5 |  |  |  |  |  |  |  |  |  |
| 11 | 63 ± 4 |  |  |  |  |  |  |  |  |  |
| 12 | 0.33 |  |  |  | 1624 |  |  |  |  |  |
| 13 | 12 |  |  |  |  |  |  |  |  |  |
| 14 | 3.3 |  |  |  |  |  |  |  |  |  |
| 15 | 3.2 |  |  |  |  |  |  |  |  |  |
| 16 | 19 |  |  |  |  |  |  |  |  |  |
| 17 | 31.62 |  |  | >10,000 |  |  |  | 3981.07 | 3981.07 |  |
| 18 | 1.258925 |  |  | >10,000 |  |  |  | 316.227766 | 2,511.89 |  |
| 19 | 1.258925 |  |  | >10,000 |  |  |  | 316.227766 | 2,511.89 |  |
| 20 | 451 |  |  |  |  |  |  |  |  |  |
| 21 | 8.69 |  |  |  |  |  |  |  |  |  |
| 22 | 74 |  |  |  |  |  |  |  |  |  |
| 23 | 0.5 |  | 0.84 |  |  | 8.5 | 30 | 18 | 20 | 17 |
| **No.** | **5-HT7** | **5-HT7a** | **adrenergic ⍺ 1** | **adrenergic ⍺ 1B** | **adrenergic ⍺ 2A** | **adrenergic ⍺ 2C** | **DOPAMINE D1** | **DOPAMINE D2** | **DOPAMINE D3** | **DOPAMINE D4** |
| 24 | 10 |  | 308 |  |  |  |  | 1033 |  |  |
| 24 | 12 |  | 278 |  |  |  |  | 1690 |  |  |
| 24 | 10 |  | 167 |  | >5,000 |  |  | 267 |  |  |
| 24 | 7 |  | 200 |  | >5,000 |  |  | 506 |  |  |
| 25 | 3 |  | 181 |  |  |  |  | 715 |  |  |
| 26 | 749 |  |  |  |  |  |  |  |  |  |
| 27 | 19 |  |  |  |  |  |  |  |  |  |
| 27 | 1.258925 |  |  | >10,000 |  |  |  | 316.227766 | 2511.886432 |  |
| 27 | 12 |  |  |  |  |  |  |  |  |  |
| 28 | 8.7 |  |  |  |  |  |  |  |  |  |
| 28 | 1.258925 |  |  | >10,000 |  |  |  | 316.227766 | 2511.886432 |  |
| 29 | 0.7 |  |  |  |  |  |  |  |  |  |
| 30 | 1.258925 |  |  | >10,000 |  |  |  | 316.227766 | 2511.886432 |  |
| 31 | 19 |  |  |  |  |  |  |  |  |  |
| 32 | 18 |  |  |  |  |  |  | 37 |  |  |
| 32 | 55 |  |  |  |  |  |  | 36 |  |  |
| **No.** | **5-HT7** | **5-HT7a** | **adrenergic ⍺ 1** | **adrenergic ⍺ 1B** | **adrenergic ⍺ 2A** | **adrenergic ⍺ 2C** | **DOPAMINE D1** | **DOPAMINE D2** | **DOPAMINE D3** | **DOPAMINE D4** |
| 33 | 32 |  |  |  |  |  |  | 328 |  |  |
| 33 | 0.5 |  | 0.84 |  |  | 8.5 | 30 | 18 | 20 | 17 |
| 34 | 77 (IC50) |  | 23 |  |  |  |  | 219 |  |  |
| 34 | 34 |  | 13 |  |  |  |  | 54 |  |  |
| 35 | 34 |  | 13 |  |  |  |  | 54 |  |  |
| 36 | 77 (IC50) |  | 23 |  |  |  |  | 219 |  |  |
| 36 | 34 |  | 13 |  |  |  |  | 54 |  |  |
| 37 | 77 (IC50) |  | 23 |  |  |  |  | 219 |  |  |
| 37 | 34 |  | 13 |  |  |  |  | 54 |  |  |
| 38 | 1.258925 |  |  | >10,000 |  |  |  | 316.227766 | 2511.886432 |  |
| 39 | 0.79 |  | 71.3 |  |  |  | 3360 | 950 |  |  |
| 39 | 7 |  | 42 |  |  |  | 2400 | 960 |  |  |
| 39 | 44 |  | 20 |  |  |  | 1900 | 330 |  |  |
| 40 | 0.79 |  | 215 |  |  |  |  |  |  |  |
| 40 | 7 |  | 42 |  |  |  |  |  |  |  |
| 41 | 25 |  |  |  |  |  |  |  |  |  |
| 41 | 35 |  |  |  |  |  |  |  |  |  |
| **No.** | **5-HT7** | **5-HT7a** | **adrenergic ⍺ 1** | **adrenergic ⍺ 1B** | **adrenergic ⍺ 2A** | **adrenergic ⍺ 2C** | **DOPAMINE D1** | **DOPAMINE D2** | **DOPAMINE D3** | **DOPAMINE D4** |
| 42 | 23 |  |  |  |  |  |  |  |  |  |
| 42 | 25 |  |  |  |  |  |  |  |  |  |
| 42 | 34 |  |  |  |  |  |  |  |  |  |
| 43 | 1.258925 |  |  | >10,000 |  |  |  | 316.227766 | 2511.886432 |  |
| 44 | 1.258925 |  |  | >10,000 |  |  |  | 316.227766 | 2511.886432 |  |
| 45 | 1.258925 |  |  | >10,000 |  |  |  | 316.227766 | 2511.886432 |  |
| 46 | 196 |  |  |  |  |  |  | 14 |  |  |
| 46 | 25 |  |  |  |  |  |  | 16 |  |  |
| 46 | 50 |  |  |  |  |  |  | 190 |  |  |
| 47 | 30 |  |  |  |  |  |  | 4 |  |  |
| 47 | 43 |  |  |  |  |  |  | 24 |  |  |
| 48 | 30.8 |  |  |  |  |  |  |  |  |  |
| 49 | 13 |  | 155 |  |  |  |  |  |  |  |
| 50 | 47 |  |  |  |  |  |  | 60 |  |  |
| 51 | 12 |  | 186 |  |  |  |  | 16 |  |  |
| 51 | 56 |  | 255 |  |  |  |  | 17 |  |  |
| 52 | 0.3 |  | 629 |  |  |  |  | 51 |  |  |
| 52 | 9 |  | 979 |  |  |  |  | 102 |  |  |
